# Nanoplasmonic Isosbestics Uncover Mesoscale Assembly of Gold Nanoparticles on Soft Templates

**DOI:** 10.1021/jacs.5c05189

**Published:** 2025-05-30

**Authors:** Jacopo Cardellini, Ilaria De Santis, Giuseppe Emanuele Lio, Marco Brucale, Francesco Valle, Virginia Catani, Ilenia Mastrolia, Marta Calabria, Massimo Dominici, Andrea Zendrini, Annalisa Radeghieri, Lucia Paolini, Paolo Bergese, Lucrezia Caselli, Debora Berti, Costanza Montis

**Affiliations:** † Department of Chemistry “Ugo Schiff”, 9300 University of Florence, Sesto Fiorentino, 50019 Florence, Italy; ‡ CSGI, Center for Colloid and Surface Science, 50019 Florence, Italy; § European Laboratory for Non-Linear Spectroscopy (LENS), Sesto Fiorentino, 50019 Florence, Italy; ∥ Department of Physics, University of Florence, Sesto Fiorentino, 50019 Florence, Italy; ⊥ Istituto per lo Studio dei Materiali Nanostrutturati, CNR, 40129 Bologna, Italy; # Laboratory of Cellular Therapy, Department of Medical and Surgical Sciences for Children and Adults, University Hospital of Modena, 41124 Modena, Italy; ∇ Division of Medical Oncology, Department of Medical and Surgical Sciences for Children and Adults, University Hospital of Modena, 41124 Modena, Italy; ○ Department of Molecular and Translational Medicine, 9297 University of Brescia, 25123 Brescia, Italy; ◆ Department of Medical and Surgical Specialties, Radiological Sciences and Public Health, University of Brescia, 25123 Brescia, Italy

## Abstract

Assembly of plasmonic nanoparticles (NPs) generates unique optical properties through coupling of the localized surface plasmon resonance (LSPR) of individual NPs. However, precisely controlling and monitoring how mesoscale assembly dictates final optical properties remain key challenges in designing advanced plasmonic materials. Here, we introduce “nanoplasmonic isosbestics” as optical descriptors of the mesoscale organization of gold nanoparticles (AuNPs) on soft templates. Unlike isosbestic points in molecular spectroscopy, which describe chemical equilibria, our numerical simulations demonstrate that nanoplasmonic isosbestics emerge from the coexistence of individual AuNPs and AuNP clusters, where the interparticle spacing determines the isosbestic wavelength. By templating AuNP assembly onto synthetic free-standing lipid bilayers with tunable membrane rigidity, we experimentally achieve precise control over interparticle spacing and prove that it is mirrored by univocal modulation of the isosbestic wavelength. This provides a fundamental understanding of the structure–function relationship in plasmonic systems, linking, for the first time, nanoplasmonic isosbestics to interparticle spacing and equilibrium structure in plasmonic assemblies. On the analytical perspective, nanoplasmonic isosbestics provide noninvasive optical fingerprints of the templates, opening to appealing applications. As a proof of concept, we apply this approach to profile the stiffness of two extracellular vesicle (EVs) classes—mesenchymal stem cell (MSC)-derived and red blood cell-derived EVs—both recognized for their biological and translational potential.

## Graphical abstract

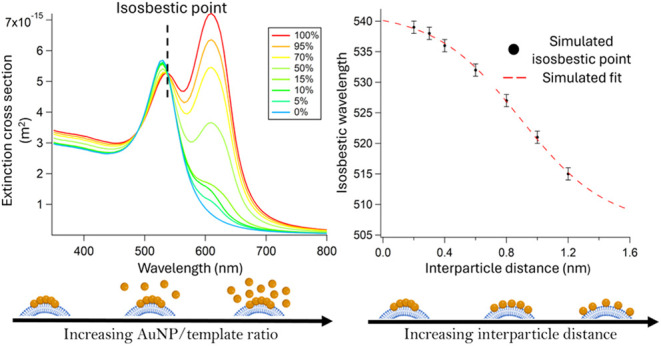

## 1 Introduction

Motivated by their unique optical properties, plasmonic nanoparticles (NPs) have attracted extensive research interest over the past decades, driving innovative applications in medicine, biosensing, catalysis, nanotechnology, and materials engineering.^1^ These properties originate from the localized surface plasmon resonance (LSPR), a phenomenon where conduction electrons coherently oscillate at the NP interface with a dielectric medium under light irradiation at resonant frequencies. Controlling such light–matter interactions at the nanoscale enables the design of materials with tailored optical properties for diverse applications. Recent advances in the synthesis of plasmonic NPs with nonspherical shapes^2,3^ (e.g., rods, cubes, cages, and stars) have extended the tunability of LSPR from the ultraviolet to the far-infrared,^4^ enabling applications in fields such as in plasmon-enhanced fluorescence,^5^ surface-enhanced Raman scattering (SERS), plasmon-enhanced photothermal and photodynamic therapies, and *in vitro* or *in vivo* bioimaging.^6–9^ The assembly of plasmonic NPs into ordered or disordered clusters further broadens the range of possibilities, offering a powerful approach to modulate and enhance LSPR, unlocking new plasmonic behaviors.^10^ Upon assembly into 1D, 2D, or 3D clusters, the LSPR modes couple and hybridize, yielding new plasmon features that depend on the cluster size, morphology, and interparticle spacing, and creating enhanced electric fields (“hot spots”) at interparticle gaps.^11–13^ The application of such structures in (bio)­sensing has enormously improved the detection limit of molecular species.^14,15^ At the same time, the emergence of lithographic technologies to fabricate highly ordered plasmonic arrays (e.g., from 2D films to 3D supercrystals) has promoted new opportunities in refractive index sensing, surface-enhanced spectroscopies, nanolasing, photocatalysis, optoelectronics, and photonics.^16–19^ Alternatively, controlled clustering of plasmonic NPs can be achieved with lower synthetic efforts, using spontaneous assembly onto soft templates in liquid media, such as liposomes,^20–26^ lipid nanoparticles,^27^ polymeric NPs,^28–30^ lipoproteins,^31^ and biological vesicles.^32^ In these cases, the structure of NP assemblies originates from thermodynamic equilibrium, and the LSPR variation induced by clustering can yield information on the physicochemical properties of the templating agent, including the concentration,^33^ nanomechanics,^32^ and purity from biological contaminants.^34^ In addition, NP assembly onto chiral soft templates (e.g., liquid crystals) can generate chiroptical properties, a recent breakthrough in the field of plasmonic NPs.^35–37^

Despite these advancements, establishing a precise relationship between the mesoscale structure of plasmonic assemblies and their optical properties remains a key challenge.^12,38^ This gap hinders the optimization of plasmonic assemblies for targeted applications and limits the development of advanced materials with new plasmonic properties, potentially unlocking transformative applications across different fields.

Here, we propose “nanoplasmonic isosbestics” as novel descriptors to elucidate the relationship between the mesostructure of NPs’ assemblies onto soft templates and their plasmonic behavior. Isosbestic points are characteristic wavelengths where the total absorbance of a system remains constant during a chemical or physical transformation.^17^ In molecular spectroscopy, isosbestic points indicate interconversion between molecular species in equilibrium chemical reactions.^39^ Conversely, isosbestic points in nanoplasmonics remains unexplored, despite several observations reported in the literature.^40,41^ Rather than molecular changes, nanoplasmonic isosbestics likely reflect morphological or structural transformations occurring at the nanoscale, potentially ranging from shape elongation to surface-ligand substitution and NPs’ self-assembly.

To demonstrate this, we use synthetic phospholipid liposomes as model soft substrates to template Turkevich–Frens AuNPs self-assembly. By combining structural and optical measurements with numerical simulations, we show that nanoplasmonic isosbestics originate from the coexistence of free AuNPs in dispersion and AuNP clusters onto the liposomal membrane. In addition, employing liposomes with different membrane rigidity, we provide evidence of a fine control over interparticle spacing (<sp>) in AuNP assemblies. This results in a systematic modulation of the isosbestic wavelength, precisely mirroring <sp>. This demonstrates, for the first time, that the plasmonic isosbestic encodes structural information on AuNP clusters, establishing it as a unique fingerprint of NP self-assembly on soft colloidal templates.

Finally, given the fact that self-assembly on soft templates occurs under thermodynamic control and that the interparticle distance is determined by the stiffness of the template, we provide a proof-of-concept that the isosbestic wavelength can be used to quantify the mechanical properties of the template. We demonstrate this for two different kinds of human extracellular vesicles (EVs), derived from red blood (RBC-EVs) and mesenchymal stem cells (MSC-EVs). This result provides additional depth to our findings, highlighting that nanoplasmonic isosbestics can represent new descriptors to characterize synthetic and biological soft materials.

## 2 Materials and Methods

### 2.1 Materials

Tetrachloroauric (III) acid (HAuCl_4_, PM = 393.83 g/mol, ≥99.9%) and trisodium citrate dihydrate (Na_3_C_6_H_5_O_7_·2H_2_O, PM = 294.10 g/mol, ≥99.9%) for the synthesis of AuNPs were provided by Sigma-Aldrich (St. Louis, MO). 1,2-Dioleoyl-*sn*-glycero-3-phosphocholine (DOPC, MW= 786.113 g/mol, >99%), 1-palmitoyl-2-oleoyl-*sn*-glycero-3-phosphocholine (POPC, MW = 760.076 g/mol, ≥98.0%), 1,2-dipalmitoyl-*sn*-glycero-3-phosphocholine (DPPC, MW = 734.039 g/mol, >99%), and 1,2-distearoyl-*sn*-glycero-3-phosphocholine (DSPC, MW = 790.145 g/mol, >99%) for liposomes’ preparation were provided by Avanti Polar Lipids (Alabaster). All chemicals were used as received. Milli-Q-grade water was used in all of the preparations.

### 2.2 Synthesis of Citrate-Capped AuNPs

We synthesized anionic citrate-capped AuNPs according to the Turkevich–Frens method.^42^ To this purpose, we prepared a solution of tetrachloroauric acid dissolving 20.0 mg in 50 mL of Milli-Q water. We then brought it to boiling temperature and added a citric acid solution (76.0 mg in 5 mL of Milli-Q water) under stirring. The dispersion was then slowly cooled to room temperature. AuNPs were characterized by the parameters described in Table 1 (see Section S2 for full AuNP characterization).

**Table 1:** Physicochemical Parameters Describing AuNPs, i.e., Core Radius (*R*_core_), Hydrodynamic Diameter (*D*_h_), Surface ζ*-*Potential, and Molar Concentration

	*R*_core_ (nm)	PDI	*D*_h_ (nm)	ζ*-*potential (mV)	concentration
AuNPs	6.9	0.130	20 ± 1	–35 ± 3	7.5·10^–9^ M (4.5·10^12^ particles/mL)

### 2.3 Preparation of Liposome Templates

DOPC, DOPC/POPC (50/50 mol %), POPC, POPC/DPPC (50/50 mol %), and POPC/DSPC (50/50 mol %) vesicles were produced according to a thin-film hydration protocol.^43^ To this purpose, we dissolved lipids in CHCl_3_ and evaporate the solvent through N_2_ flux and overnight vacuum drying to obtain a dry lipid film onto the bottom wall of a glass vial. We then hydrated the film with Milli-Q water at 50 °C, reaching a final lipid concentration of 4 mg/mL (i.e., 1·10^13^ particles/mL, see Section S1.1). Through this procedure, we obtained multilamellar vesicles that we subsequently subjected to ten freeze and thaw cycles.^2^ To reduce the polydispersity,^3^ we finally extruded the vesicles through polycarbonate membranes (Nuclepore Track-Etch Membrane, Whatman, Cytiva, Buckinghamshire, UK) with pores diameter of 100 nm 10 times at 50 °C, employing an extruder (Lipex Biomembranes, Vancouver, Canada). Different liposome dispersions were characterized by the parameters described in Table 2 (see also Section S1 for a full characterization).

**Table 2:** Physicochemical Parameters Describing Liposomes, i.e., Hydrodynamic Diameter (*D*_h_), Polydispersity (PDI), Surface ζ*-*Potential, and Stiffness (Measured with Atomic Force Microscopy (AFM); see Section 2.11)

sample name	composition (mol %)	*D*_h_ (nm)	PDI	ζ*-*potential (mV)	stiffness (N/m)
DOPC	100	103 ± 2	0.041 ± 0.025	0 ± 3	0.006 ± 0.001
DOPC/POPC	50/50	116 ± 4	0.058 ± 0.039	–3 ± 3	0.011 ± 0.003
POPC	100	106 ± 1	0.112 ± 0.040	–4 ± 2	0.012 ± 0.002
POPC/DPPC	50/50	108 ± 1	0.033 ± 0.017	–4 ± 2	0.018 ± 0.002
POPC/DSPC	50/50	134 ± 11	0.114 ± 0.032	–5 ± 1	0.031 ± 0.006

### 2.4 Red Blood Cell-Derived Extracellular Vesicles (RBC-EVs) and Mesenchymal Stem Cell-Derived Extracellular Vesicles (MSC-EVs) Production and Characterization

RBC-EVs and MSC-EVs were produced as described in detail in Section S5. Briefly, RBC-EVs were separated by differential centrifugation of a suspension of red blood cells induced by calcium ionophore following protocols previously reported.^44^ MSC-EVs were separated from a MSC-conditioned medium with Tangential Flow Filtration processing following the protocol described in Section S5.1. Both RBC-EV and MSC-EVs were characterized as described below and in Section S5.2-4.

### 2.5 Bicinchoninic Acid (BCA) Assay

Protein concentrations of RBC-EV and MSC-EV samples were determined with Pierce BCA Protein Assay Kit (Thermo Fisher, Rockford) following manufacturer’s instructions.

### 2.6 Nanoparticle Tracking Analysis (NTA)

Nanoparticle tracking analysis (NTA) was conducted following manufacturer’s instructions, employing a NanoSight NS300 system (Malvern Panalytical, Malvern, UK) equipped with a 532 nm laser. To achieve an optimal particle per frame value (ranging from 20 to 100 particles per frame), samples were diluted at a 1:1000 ratio in filtered PBS. A syringe pump was used for constant flow injection at 20 μL/min, while the temperature was maintained at a constant 25 °C. The particles were detected with a camera level set at 10, and three videos, each lasting 60 s, were captured and subsequently analyzed using NTA software version 3.2. The mean, mode, and median sizes of the EVs from each video were utilized to calculate the sample concentration, which was expressed in particles per milliliter (particles/mL).

### 2.7 Preparation of Vesicles-AuNP Assemblies

To detect plasmonic isosbestic points, a proper amount of liposomes in Milli-Q was incubated for 5 min at room temperature with AuNPs, to achieve a final AuNP concentration of 4.5·10^12^ particles/mL. Based on a previous study,^20^ we employed a 5 min incubation, which is sufficient to ensure the formation of equilibrium AuNP cluster structures while remaining short enough to prevent sample flocculation and precipitation, caused by the formation of AuNP “bridges” connecting multiple liposomes. Different concentrations of liposomes in the final mix were used (in the range of 5.987·10^10^–6.545·10^11^ particles/mL). Corresponding samples containing RBC-EVs and MSC-EVs were prepared according to the same protocol, employing EVs in the concentration range of 2·10^9^–8·10^9^ particles/mL. Samples were diluted by the addition of 700 μL of Milli-Q water before measurements. For Cryo-EM and small-angle X-ray scattering (SAXS) characterization, a high concentration of liposomes (3.2·10^11^ particles/mL in the final mix) was employed to maximize AuNP clustering, corresponding to a liposomes/AuNPs number ratio of ∼1/14.

### 2.8 UV–Vis Spectroscopy

We recorded UV–vis spectra using a Cary 3500 Multizone UV–vis spectrophotometer (Agilent Technologies Inc., Santa Clara, CA), equipped with a xenon lamp, emitting a radiation of 250 Hz frequency in the 190–1100 nm wavelength range. The radiation is transmitted through an optical fiber to reach eight different sample positions, each one having its own detector. We placed samples (prepared as described in Section 2.7) in poly­(methyl methacrylate) cuvettes (BRAND semimicro cuvettes, BRAND GmbH + Co. KG, Wertheim, Germany). Then, we acquired UV–vis spectra in the 350–800 nm range of wavelengths. Each spectrum was normalized at 350 nm to minimize eventual concentration differences due to sample preparation.

### 2.9 Small-Angle X-Ray Scattering (SAXS)

We performed Synchrotron SAXS measurements at beamline ID02 at the European Synchrotron Radiation Facility (ESRF, The European Synchrotron, 71 Avenue des Martyrs, CS40220, 38043 Grenoble Cedex 9)^−1^. Employing a sample–detector distance of 2 m and a single-beam setting for monochromatic X-ray radiation of 0.1 nm wavelength (12.23 keV), we covered a scattering vector (*Q*) range of 035 ≤ *Q* ≤ 4.218 nm^–1^. The detector was a 2D Rayonix MX-170HS with a pixel size of 44 × 44 μm^2^. Samples were measured in glass capillaries of 1.5 mm thickness. 2D scattering patterns were normalized to an absolute intensity scale, based on transmission and sample thickness, and then azimuthally averaged to generate 1D intensity profiles (*I*(*Q*)). These profiles were analyzed employing the SasView software (https://www.sasview.org/).

### 2.10 Cryogenic Electron Microscopy (Cryo-EM)

Cryo-EM imaging was performed at the Florence Center for Electron Nanoscopy (FloCEN), University of Florence, through a Glacios (Thermo Fisher Scientific) instrument operating at 200 kV equipped with a Falcon III detector. Regarding sample preparation, we deposited 3 μL of vesicles-AuNP hybrids on glow-discharged Quantifoil Cu 300 R2/2 grids. Employing a FEI Vitrobot Mark IV (Thermo Fisher Scientific), we plunge-freezed the grids in liquid ethane, followed by 1 s blotting with filter papers at 100% humidity and 10 °C. Finally, we acquired images using EPU software with 2.5 Å pixel size and total electron dose of ∼50 e^–^/Å^2^ per micrograph. Image analysis was performed with ImageJ software.

### 2.11 Atomic Force Microscopy (AFM)

AFM imaging, quantitative morphometry, and single-particle nanoindentation were performed as described elsewhere.^45,46^ Briefly, all samples were deposited on poly-l-lysine (PLL)-coated glass coverslips and scanned in Milli-Q water at room temperature on a Bruker Multimode 8 microscope equipped with a Nanoscope V controller, a sealed fluid cell, and a type JV piezoelectric scanner using Bruker SNL-A probes calibrated with the thermal noise method. Image background subtraction was performed using Gwyddion 2.61.^47^ The mechanical characterization of individual vesicles was performed by either measuring their stiffness via single-particle nanoindentation or by measuring their surface contact angle.^46,48^

### 2.12 Dynamic Light Scattering (DLS) and ζ-Potential

We performed DLS and ζ-potential using a Brookhaven Instrument 90 Plus (Brookhaven, Holtsville, NY) operating at θ = 90°. DLS measurements were obtained as averages of 10 repetitions of 1 min each. The autocorrelation functions were analyzed through the CONTIN algorithm.^49^ ζ*-*Potential values were obtained as averages of 10 measurements and calculated from the electrophoretic mobility *u*, through the Helmholtz–Smoluchowski equation: ζ = (η/ε) × *u*, where η is the viscosity and ε is the dielectric permittivity of the dispersing medium.

### 2.13 Numerical Methods

We calculated the absorption and scattering cross sections in the electromagnetic wave, frequency domain (emw) module on COMSOL Multiphysics, through the following equations

1 $$\sigma_{\mathrm{abs}}=W_{\mathrm{abs}}/P_{\mathrm{in}}$$

2 $$\sigma_{\mathrm{sca}}=W_{\mathrm{sca}}/P_{\mathrm{in}}$$

where *P*_in_ is the incident irradiance, defined as energy flux of the incident wave; *W*_abs_ is the energy rate absorbed per particle, derived by integrating the energy loss over the volume of the particle, while *W*_sca_ is the energy rate scattered per particle, derived by integrating the Poynting vector over an imaginary sphere around the particles. The extinction cross section was calculated as the sum of the absorption and scattering cross sections

3 $$\sigma_{\mathrm{ext}}=\sigma_{\mathrm{abs}}+\sigma_{\mathrm{sca}}$$

Other parameters, i.e., the scattered field method, proper polarization, and propagation direction, were opportunely set to numerically investigate the near-field distribution and plasmonic response of AuNPs. The light beam intensity was evaluated through the following equation

4 $$I=\left({E_{0}}^{2}\right)/\left(2\cdot Z_{0\;\mathrm{const}}\right)=1.33\cdot 10^{7}\;W/m^{2}$$

where *E*_0_ is the initial electric field (in our case 1·10^5^ V/m) and *Z*_0 const_= 376.73 Ω is the impedance of the system, also considering the incident area. This COMSOL Multiphysics tool package offers the possibility of studying the energy flow that passes through AuNPs when they are shone by a light beam and the energy flow scattered from AuNPs and collected on the external layer used as an integrating sphere. To closely simulate the behavior observed in the experiments, the numerical system was designed as follows: a total of 63 AuNPs was considered, with AuNPs placed at a defined interparticle distance (sp). This geometrical parameter refers to the distance between two AuNP surfaces (i.e., edge-to-edge distance) (Figure 1A). To introduce structural variability and account for the uncertainty in the interparticle distance, we grouped the total 63 AuNPs in 21 subgroups, each consisting of three AuNPs with an average interparticle distance denoted as <sp> (Figure 1B). <sp> was obtained considering the input interparticle distance sp, and its variations, i.e., sp^–^ = sp – sp/4 and sp^+^ = sp + sp/4. This approach allowed us to model a system where each AuNP subgroup could adopt slightly different interparticle distances—sp, sp^–^, or sp^+^—thus introducing realistic structural variability. By varying the percentage of AuNPs that are close or far to each other, the obtained plasmonic resonances resulted as the convolution of the ones for each single interparticle distance.

**Figure 1:**
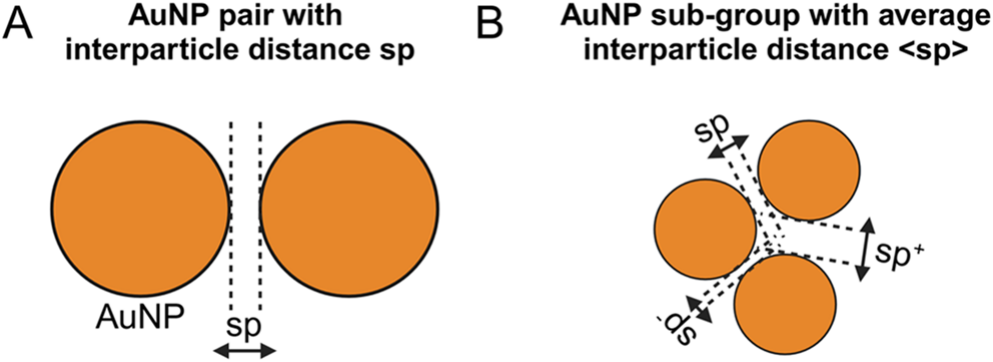
(A) Schematic representation of an AuNP pair with a surface-to-surface interparticle distance sp and (B) AuNP subgroup consisting of three AuNPs placed at different interparticle distances (sp, sp^+^, and sp^–^), resulting in an average interparticle distance of <sp>.

The materials involved in these simulations are Au for the NPs, and liposomes are the surrounding material. For each one, in the materials section of the software, the following data were considered:

- Au: real and imaginary part of permittivity as a function of wavelength from the materials library of the software itself, permeability μ = 1, and electrical conductivity σ = 0 (S/m);
- liposomes: real part of permittivity fixed to 1.343, permeability μ = 1, and electrical conductivity σ = 0 (S/m).

### 2.14 Statistical Analysis

Data of Figure 5H have been statistically analyzed using GraphPad Prism 10.4.0. Statistical significance (*P* values) was calculated using ordinary one-way ANOVA followed by Tukey’s multiple comparisons test to compare experimental isosbestic wavelengths for the different liposome-AuNP assemblies: *P* > 0.05 (ns, nonsignificant), *P* < 0.05 (*), *P* < 0.01 (**), *P* < 0.001 (***), and *P* < 0.0001 (****). A familywise α threshold and a confidence level of α = 0.1 (90% confidence interval) were used.

## 3 Results

### 3.1 AuNP Clustering onto Liposomal Templates: Emergence of Nanoplasmonic Isosbestics

To investigate the plasmonic properties of AuNP assemblies, we selected a simple and highly controlled system, i.e., clusters of citrate-stabilized AuNPs confined onto the surface of zwitterionic free-standing lipid membranes. Such systems form spontaneously in aqueous dispersions when AuNPs interact with synthetic or natural vesicles, leading to ordered clusters whose structure strongly depends on the membrane’s physical chemistry.^20,24^

Figure 2A shows Cryo-EM imaging for ∼12 nm citrate-stabilized AuNPs (Table 1) after incubation with DOPC unilamellar liposomes with a diameter of ∼100 nm and close to neutral surface charge (Table 2) in Milli-Q water. As shown, AuNPs form a compact cluster on the vesicle surface, associated with a rapid color change of the dispersions from red to purple-blue due to AuNP–AuNP plasmon coupling. Figure 2B displays the UV–vis spectra of AuNPs at a fixed concentration of 4.4·10^12^ particles/mL incubated with DOPC vesicles at varying concentrations in the range of 6·10^10^–3.2·10^11^ particles/mL (see Section 2.7 for sample preparation). Of note, AuNPs are in large excess compared to vesicles across the whole concentration range (i.e., from ∼74/1 to ∼14/1 AuNPs/liposomes number ratio). Low vesicles concentrations induce negligible variations in the plasmonic signal of original AuNPs due to the limited template’s area available for AuNP clustering, leaving the vast majority of AuNPs dispersed in the medium. Increasing the vesicle concentration alters the plasmonic signal of AuNPs, inducing a progressive broadening of the primary LSPR peak. This indicates that a progressively broader AuNP population is involved in clustering, culminating in the emergence of a new distinct red-shifted plasmonic peak with a wavelength of ∼603.5 nm, marking interparticle plasmonic coupling.

**Figure 2:**
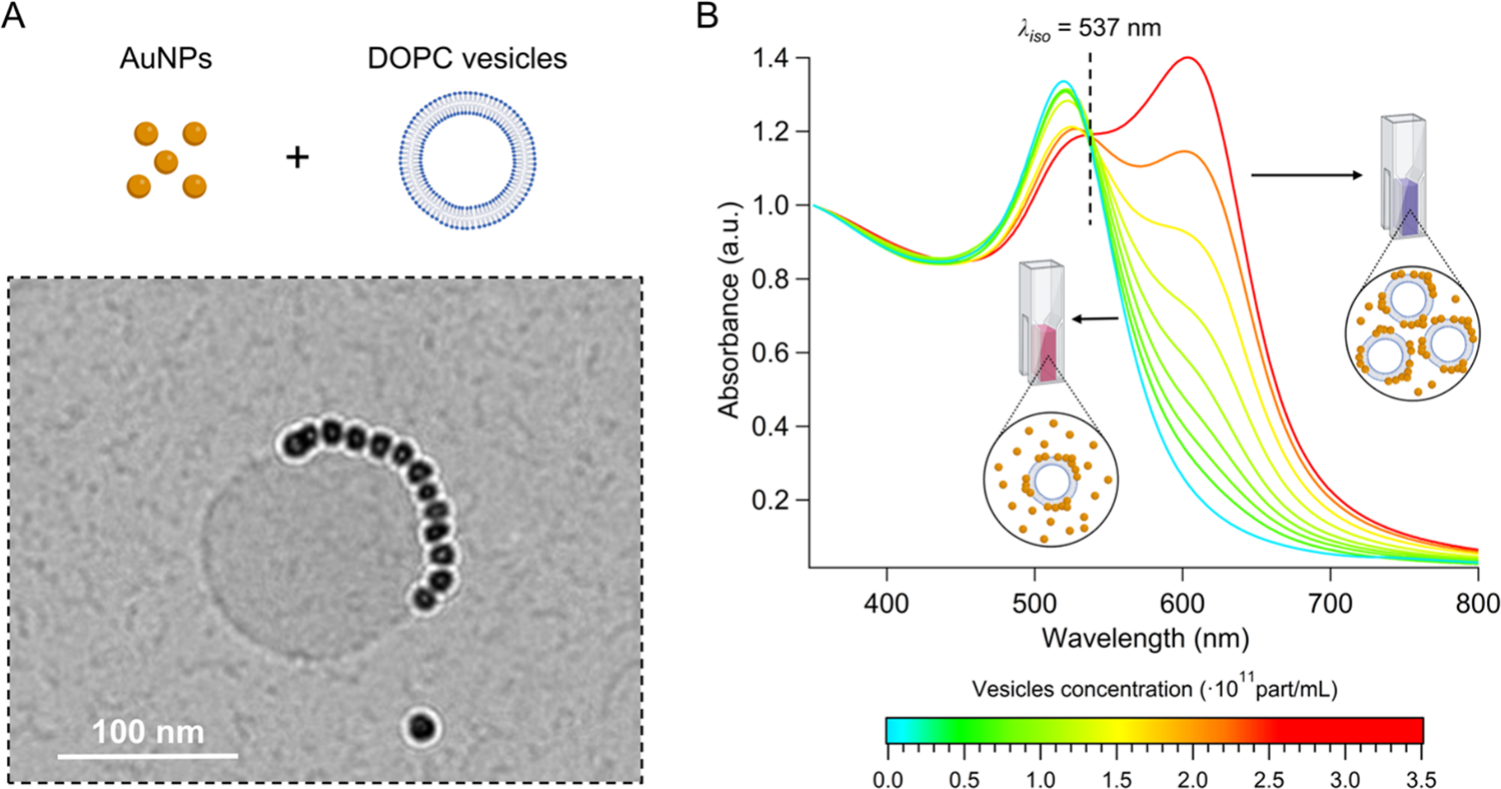
(A) Representative Cryo-EM images of AuNPs (4.4·10^12^ particles/mL) interacting with DOPC liposomes (3.2·10^11^ particles/mL) in Milli-Q water (see Figure S6 for additional images) and (B) UV–vis spectra of AuNPs (4.4·10^12^ particles/mL) incubated with DOPC liposomes at different concentrations (6·10^10^–3.2·10^11^ particles/mL). The dashed line indicates the isosbestic wavelength (λ_iso_). Elements of this figure were created in BioRender. Zendrini, A. (2025) (https://BioRender.com/308kucw).

Plasmonic coupling introduces another distinctive feature: the spectra of AuNPs at varying liposome concentrations intersect at a specific wavelength (i.e., 537 nm), known as the *isosbestic point*. This is a characteristic wavelength where the absorbance remains invariant despite variations in the vesicles’ concentration. In spectroscopy, isosbestic points identify the copresence of two (or more) molecular species during chemical reactions (e.g., conversion of reagents into products), sharing the same molar absorption coefficient at a given wavelength.^50–52^ For this phenomenon to occur, the reaction must proceed with constant stoichiometry and be free from secondary reactions or side products. Here, the “nanoplasmonic isosbestic” arises from the self-assembly of AuNPs upon interaction with liposomes, reflecting transformations occurring at the mesoscale rather than at the molecular level. In analogy to the molecular case, this spectral feature might indicate that clustering proceeds through direct evolution (i.e., not involving intermediates) of individual particles into a precise type of cluster (i.e., with well-defined structural, hence plasmonic, features). Supporting this, a few previous studies have observed plasmonic isosbestic during the temporal evolution from one plasmonic species to another and have attributed them to the equilibrium between two plasmonic species during kinetic transformations.^40,41^

However, in contrast to these previous works, the plasmonic isosbestic observed here occurs under “static conditions” in systems that have already reached a stable structural configuration. This stability is achieved by incubating liposomes with AuNPs for 5 min prior to spectral recording (see Section 2.7), a time significantly longer than the ∼30-s kinetics required for AuNP self-assembly on liposomes.^20^ In this approach, the isosbestic point arises from different structurally stable samples prepared at increasingly higher concentrations of vesicles. Increasing the template’s concentration enhances the area available for AuNP clustering, thereby modulating the relative abundance of individual and clustered AuNP populations. We hypothesized that the coexistence of these two plasmonic species at varying ratios originates from the nanoplasmonic isosbestic. To validate this hypothesis, we performed numerical simulations as described in the following section.

### 3.2 Determination of Isosbestic Species

To understand the origin of the nanoplasmonic isosbestic in the UV–vis spectra of AuNPs-vesicles and identify the associated plasmonic species, we performed numerical simulations.

Based on previous discussion (see Section 3.1), we modeled the system considering two plasmonic populations: individual AuNPs and AuNP clusters with defined and homogeneous structural features. The presence of a population of individual AuNPs was considered to simulate the excess of AuNPs compared to lipid vesicles in the concentration range where the isosbestic point is observed. To model AuNP clusters, we explored the effects of (i) interparticle spacing (<sp>) and (ii) the fraction of AuNPs in clusters. We evaluated the absorption, scattering, and extinction cross sections for isolated 12 nm spherical AuNPs and AuNP clusters with varying <sp>, and different ratios of clustered to singly dispersed AuNPs. To mimic experimental AuNP clustering on liposomes, we arranged AuNPs in clusters with a radial isotropic distribution on the *XY* plane, placed into a spherical system to emulate an integrating sphere able to collect the scattered light from all directions. This radial configuration is inherently insensitive to the polarization direction of the incident light (i.e., *Ex* = *Ey* = *Ej*, where *j* represents any polarization angle within the *XY* plane). This feature makes the 2D radial model a reliable approximation of experimental conditions, where spectra are acquired under unpolarized illumination. In addition, it significantly reduces computational costs by eliminating the need to average over separate simulations for *Ex* and *Ey* polarizations to approximate unpolarized light, as it inherently satisfies the condition *Ex* = *Ey*. This numerical setup is reported in Figure 3A, with the adopted numerical method validated in previous studies.^53–56^

**Figure 3:**
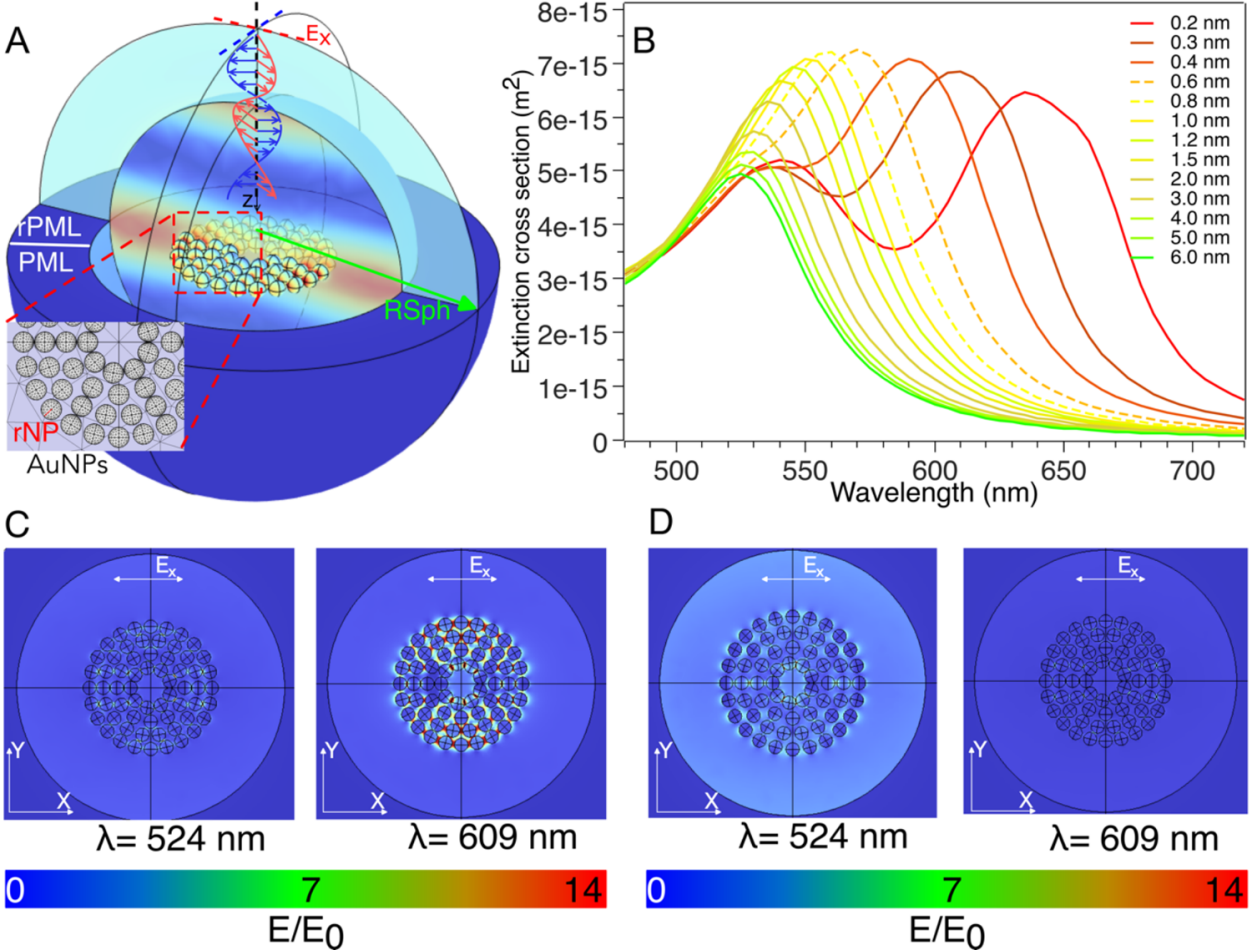
(A) Schematic illustration of the numerical simulation adopted to investigate the plasmonic properties of AuNPs arranged onto liposomal templates. The numerical simulations involve AuNPs with a radius rNP, a spherical environment with a radius RSph that has an empty inner core, and an outer layer that constitutes the perfect matched layer (PML) characterized by an outer radius rPML used to avoid multiple light scattering. The impinging light is polarized along the *X* axis. (B) Extinction cross sections evaluated using numerical simulations considering a radial arrangement of AuNPs on the *XY* plane and placed at different values of <sp>. (C, D) Electric field distribution maps calculated at two different wavelengths, i.e., λ = 524 and 609 nm and referred to the case of <sp> 0.3 nm (C) and <sp> 4 nm (D), showing the in and out of the resonance case.

To investigate the dependence of AuNP extinction cross section on <sp> within clusters, we systematically varied the spacing of the whole AuNP population to cover a wide range of distances, from subnanometer to a few nanometers (Figure 3B). In the 3–6 nm interval of <sp>, the profile of the extinction cross section resembles that of individual (plasmonically uncoupled) AuNPs, featuring a single peak centered at λ = 525 nm. As <sp> decreases, the primary plasmonic peak broadens and eventually splits into two distinct plasmonic peaks for <sp> = 0.6 nm (dashed line). Further shortening of the interparticle distance leads to a progressive red shift of the secondary plasmonic peak. These results remark the strong dependence of the AuNP plasmonic features on the interparticle spacing. The case study that most closely aligns with the experimental results of Figure 2B occurs at <sp> = 0.3 nm. For this spacing, the plasmonic profile exhibits two distinct peaks cantered at λ = 524 and 609 nm, closely matching the experimental values. Electric field distribution maps further support these findings (Figure 3C,D). At <sp> = 0.3 nm, overlapping dipoles are observed at both resonant wavelengths (λ = 609 and 524 nm) (Figure 3C). Conversely, at <sp> = 4 nm, the electric field is enhanced only at the primary resonant wavelength (λ = 524 nm), characteristic of a single-particle resonance (Figure 3D).

To assess the effect of varying ratios of dispersed to clustered AuNPs, we simulated the spectral response of a system consisting of plasmonically coupled AuNPs (forming clusters) and plasmonically uncoupled AuNPs (dispersed as single particles). We varied the ratio between these two populations while maintaining a fixed average <sp> of 0.3 nm within clusters. Changes in the proportion of coupled to uncoupled AuNPs affect the extinction cross section (Figure 4A). When AuNP coupling is complete (100% of plasmonically coupled AuNPs), the secondary plasmonic peak at λ = 609 nm reaches its maximum intensity. As the fraction of coupled AuNPs decreases in favor of uncoupled AuNPs, the intensity of this peak progressively diminishes, eventually disappearing at 0% coupled AuNPs. Under this condition, the extinction spectrum exhibits a single plasmonic peak at λ = 524 nm, characteristic of fully uncoupled AuNPs. Interestingly, numerical simulations reveal an isosbestic point at λ = 537 nm, in excellent agreement with the experimental data in Figure 2B. The corresponding absorption and scattering cross sections obtained from the numerical model are reported in Figure S11A,B. Furthermore, the isosbestic behavior observed with the 2D radial model adopted in this study was also confirmed using a different simulation setup, i.e., 1D chain-like clusters replicating the linear AuNP arrangement shown in Figure 2A (Figure S12). Given that this configuration is not isotropic in the *XY* plane, simulations were performed using two different light polarization directions (i.e., *Ex* and *Ey*) to mimic experimental unpolarized light conditions. These simulations demonstrated that the isosbestic wavelength remains invariant with respect to the cluster shape—being identical for both 2D radial and 1D chain-like clusters—and depends solely on the interparticle spacing.

**Figure 4:**
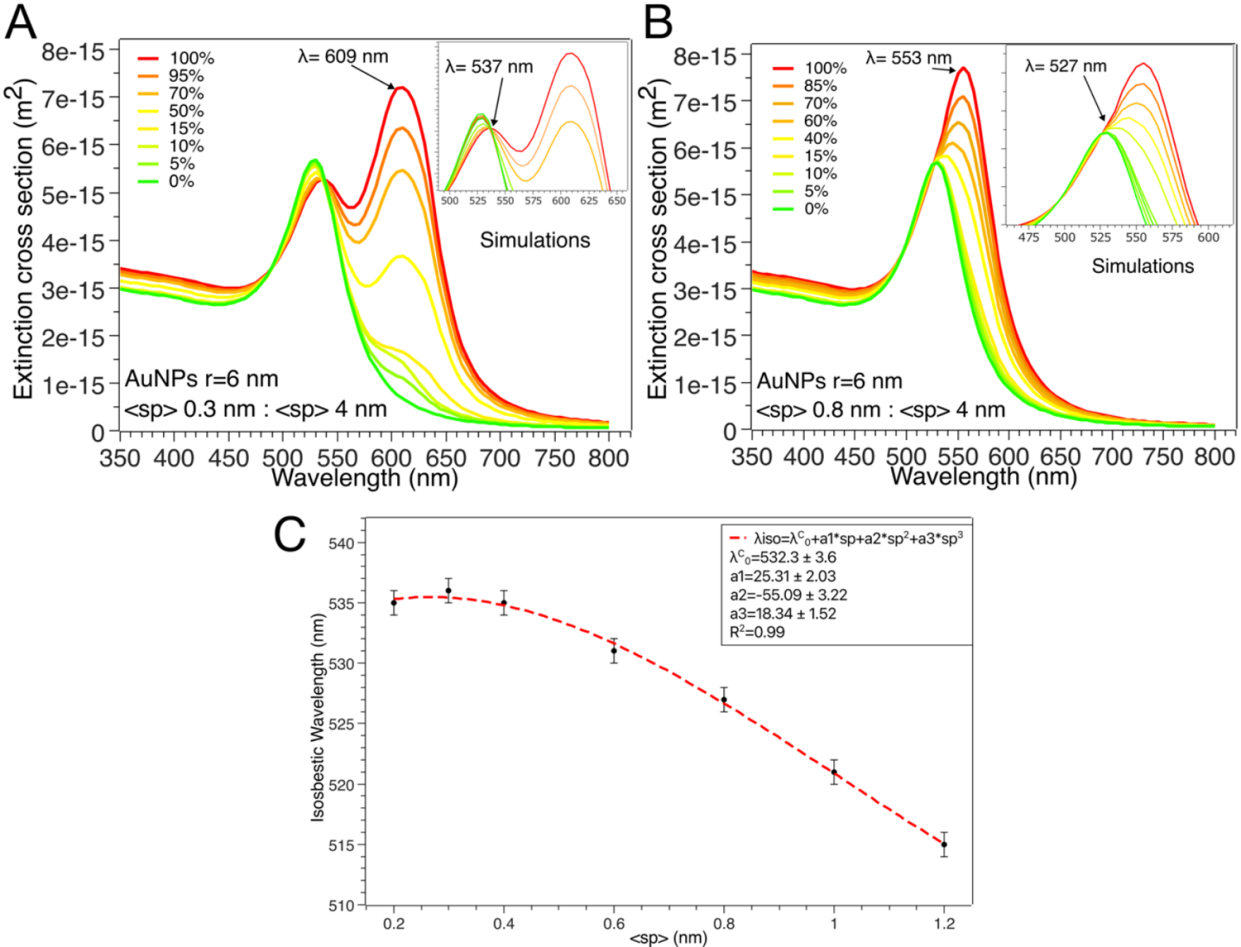
(A, B) Numerical simulations displaying the extinction cross section of AuNPs obtained by varying the percentage of individual (uncoupled) AuNPs (<sp> 4 nm) and plasmonically coupled AuNPs, with an <sp> of (A) 0.3 nm and (B) 0.8 nm; in both plots, the red line remarks the complete coupled condition (100% of plasmonically coupled of AuNPs), while the green line highlights the fully uncoupled condition (0% of plasmonically coupled AuNPs). The isosbestic wavelength is 537 nm in panel (A), while it is 527 nm in panel (B); (C) λ_iso_ values obtained from numerical simulations as a function of <sp>, fitted according to a sigmoidal law described by the parameters indicated in the graph.

We further investigated the influence of interparticle distance on the isosbestic wavelength (λ_iso_) considering a larger <sp> in AuNP clusters, i.e., 0.8 nm (Figure 4B). In this case, numerical simulations reveal a broad resonance at λ = 553 nm for 100% coupled AuNPs, that is gradually blue-shifted, decreasing the percentage of coupled AuNPs (the corresponding absorption and scattering cross sections are reported in Figure S13A,B). Remarkably, the increase in <sp> from 0.3 to 0.8 nm corresponds to a 10 nm blue shift in λ_iso_, i.e., from 537 to 527 nm.

Building on these findings, we extended our investigation across a broader range of average interparticle distances (<sp> from 0 to 1.6 nm), systematically tracking the evolution of λ_iso_ as a function of <sp>.

The simulated isosbestic wavelengths as a function <sp>, obtained from numerical simulations, are shown in Figure 4C. This trend follows a sigmoidal dependence, where λ_iso_ systematically increases as <sp> decreases

5 $$\lambda_{\mathrm{iso}}=\frac{\left(\lambda_{\mathrm{iso}}^{A}-\lambda_{\mathrm{iso}}^{B}\right)}{1+e^{\left(<\mathrm{sp}>-{\mathrm{sp}}_{0}\right)/k}}+\lambda_{\mathrm{iso}}^{B}$$

where λ_iso_^A^ and λ_iso_^B^ are fitting parameters, representing the upper limit and lower limit of the sigmoidal function, respectively, and sp_0_, i.e., the inflection point derived from fitting, marks the midpoint of the sigmoidal curve, where the isosbestic wavelength shifts most rapidly, and *k* is the slope factor determining the steepness of λ_iso_ shifts with <sp>.

Remarkably, eq 3 highlights that, if λ_iso_ is known (e.g., from UV–vis characterization), <sp> can be easily estimated.

The sigmoidal behavior is characteristic of plasmonic systems where nanoparticle interactions are regulated by distance-dependent coupling effects.^32,57^ A similar behavior was also observed for AuNPs with larger diameters, as detailed in Section S4.2, further demonstrating the general validity of our findings and the robustness of the proposed numerical approach for future studies.

Overall, our simulations confirm that nanoplasmonic isosbestics arise from the equilibrium between two plasmonic species, individual AuNPs and AuNP clusters, with well-defined interparticle spacing. A key implication is that variations in the average AuNP spacing directly modulate the isosbestic wavelength, potentially unlocking new opportunities to predict the structure of AuNP clusters based on their plasmonic properties.

These results also provide a mechanistic explanation of the UV–vis spectra in Figure 2B. According to the simulations, the experimental isosbestic point results from an increase in the number of AuNPs participating in clusters as the vesicle concentration rises. Moreover, the sigmoidal trend in Figure 4C allows for a direct estimation of interparticle distance from the experimental isosbestic wavelength, yielding a value of ∼0.3 nm for the AuNP–DOPC liposome system (Figure 2B).

### 3.3 Nanoplasmonic Isosbestics on Liposomal Templates of Varying Stiffness

To experimentally validate the dependence of the isosbestic wavelength on <sp>, we used liposomes with different rigidities as soft templates. The stiffness of lipid vesicles, i.e., their mechanical response to applied deformation,^58^ is known to affect the compactness of AuNPs clusters, offering a straightforward way to control <sp> with high precision.^20,32^ To achieve this, we prepared a series of phosphatidylcholines (PC)-based unilamellar liposomes with similar size and surface charge (see Section S1 for characterization). By varying the PC composition (Table 2), we modulated the membrane stiffness within the range of interest for biological membrane-limited systems (0.006 ± 0.001–0.031 ± 0.006 N/m). The stiffnesses of synthetic vesicles were assessed through AFM-based force spectroscopy (AFM-FS, Figure S2). When challenged with AuNPs, clusters formed with a progressively increasing <sp> as membrane stiffness increased, as shown in Cryo-EM micrographs (Figure 5A,B) comparing two extremes of liposome stiffnesses, i.e., DOPC (low stiffness, 0.006 ± 0.001 N/m) and POPC/DPPC (high stiffness, 0.018 ± 0.002 N/m) (additional Cryo-EM images are reported in Section S3.1). Cryo-EM image analysis (Figure 5A,B) allowed quantifying variations in <sp> across the vesicle set, which was found to range from 0.8 ± 0.2 nm (lowest stiffness) to 2.4 ± 0.9 nm (highest stiffness). Of note, these values fall within the <sp> interval explored in numerical simulations (from 0.2 to 6 nm).

**Figure 5:**
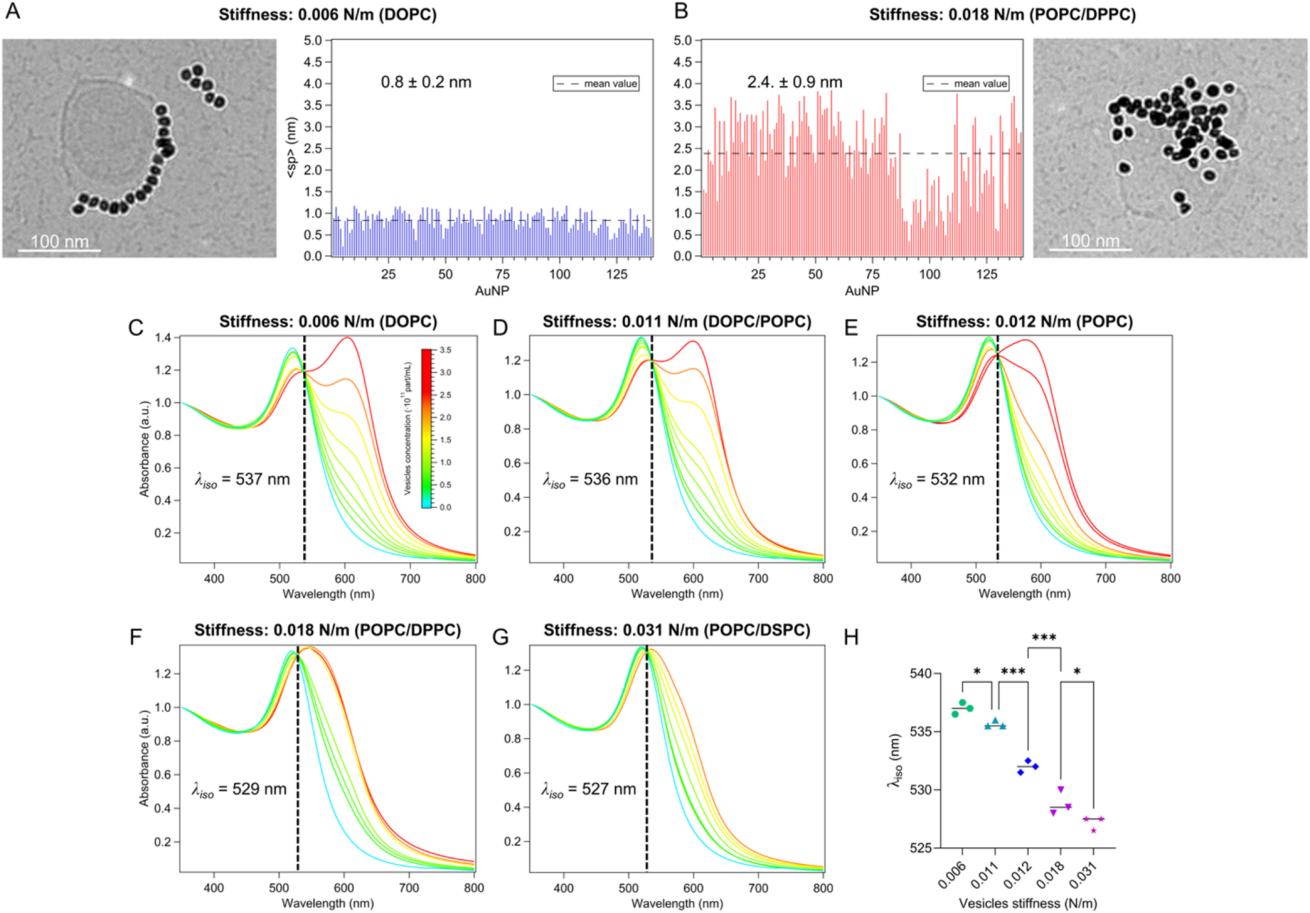
(A, B) Representative Cryo-EM images of AuNPs (4.4·10^12^ particles/mL) interacting with soft DOPC and rigid POPC/DPPC liposomes (3.2·10^11^ particles/mL) in Milli-Q, together with corresponding sp distributions for AuNP clusters, extracted from image analysis using ImageJ.^8,9^ Black dashed lines indicate the <sp>, i.e., 0.8 ± 0.2 nm for DOPC and 2.4 ± 0.9 for POPC/DPPC; (C–G) UV–vis spectra of AuNPs (4.4·10^12^ particles/mL) incubated with liposomes of varying stiffness in the concentration range of 6·10^10^–3.2·10^11^ particles/mL. (H) λ_iso_ for different liposome-AuNP assemblies vs membrane stiffness. Statistical significance was calculated as described in Section 2.14.

We then incubated AuNPs with different liposome formulations, varying the liposome concentration in the same range previously used for DOPC vesicles (Figure 2B). Plasmonic changes were monitored with UV–vis spectroscopy, with the results presented in Figure 5C–G. For each PC composition, an increasing vesicle concentration leads to enhanced plasmonic shifts, consistent with the trend observed for DOPC (Figure 2B). Additionally, plasmonic variations also increase as the vesicle stiffness decreases. Notably, an isosbestic point is present across all vesicle compositions within the selected concentration range (see Table S5 for λ_iso_ values). Moreover, the isosbestic wavelength is specific to each lipid composition, with experimental values in Figure 5C,G closely matching the simulated values for the limit cases shown in Figure 4A,B, respectively. In line with simulations, the isosbestic wavelength is finely modulated by variations in stiffness, directly corresponding to changes in <sp> (Figure 5H). Specifically, λ_iso_ decreased steadily with increasing <sp>, ranging from 537 ± 1 nm on the softest vesicles (i.e., stiffness of 0.006 ± 0.001 N/m, producing the smallest spacing) to 527 ± 1 nm on the stiffest liposomes (i.e., stiffness of 0.031 ± 0.006 N/m, yielding the largest spacing).

### 3.4 Structure of AuNP Clusters and Energetics of AuNP Interactions

To understand interaction forces governing variations in <sp> within clusters, we performed high-resolution small-angle X-ray scattering (SAXS) experiments at the European Synchrotron Radiation Facility (ESRF, Grenoble, France). These experiments provided a structural characterization of AuNP clusters as a function of vesicle stiffness, obtained at a fixed concentration of vesicles (3.2·10^11^ particles/mL) and AuNPs (4.4·10^12^ particles/mL), at which AuNPs show maximal aggregation. Under these conditions, the scattering profile is dominated by AuNPs, with a negligible scattering contribution from vesicles (see Figure S3).

Figure 6A shows the SAXS profiles of AuNPs-liposomes in the *Q* range of interest (0.03 ≤ *Q* ≤ 0.3 Å^–1^). This intermediate-high *Q* range was chosen to avoid the scattering interference from large size objects (AuNP clusters), which are captured at lower *Q* values (see Section S3.3 for corresponding SAXS profiles acquired in a wider *Q* range).^59^ Instead, this range selectively probes finer structural features, including the shape and size of individual AuNPs (encoded in the form factor (*P*(*Q*)) of AuNP cores) and the interparticle distance within clusters contained in the interparticle structure factor, i.e., *S*(*Q*). The right inset in Figure 6A displays *S*(*Q*) extracted from SAXS profiles, as described in Section S3.3. In the absence of lipid vesicles (black profile), *S*(*Q*) = 1 across the entire *Q* range, indicating well-dispersed, noninteracting AuNPs. In contrast, the emergence of *S*(*Q*) peaks upon vesicle addition indicates the formation of AuNP suprastructures, with peak positions corresponding to the average interparticle distance. Consistent with numerical simulations and Cryo-EM imaging, this interparticle distance decreases with reducing vesicle stiffness, evidenced by the shift of the *S*(*Q*) peak to higher *Q* values (see Section S3.3). To investigate interparticle interactions driving this reduction in interparticle spacings, SAXS profiles were fitted using a model that combines a spherical form factor with a Schulz size distribution^60^ and a Sticky Hard Sphere (SHS) interparticle structure factor (see Section S3.3 for details).^61,62^ The latter is widely applied to describe hard particles interacting through an attractive, short-range, potential well.^61^ During fitting, the <sp> values obtained from numerical simulations, and the particle core size, determined through SAXS analysis of neat AuNPs (Section S2), were used as input parameters, as described in Section S3.3. The analysis yielded the volume fraction of the aggregates (ϕ), reflecting the compactness of clusters, and the “stickiness parameter” (τ), which reflects the strength of the attractive well between particles. Table 3 provides a comprehensive overview of the structural parameters derived from the fitting (black dashed lines in Figure 6A).

**Figure 6:**
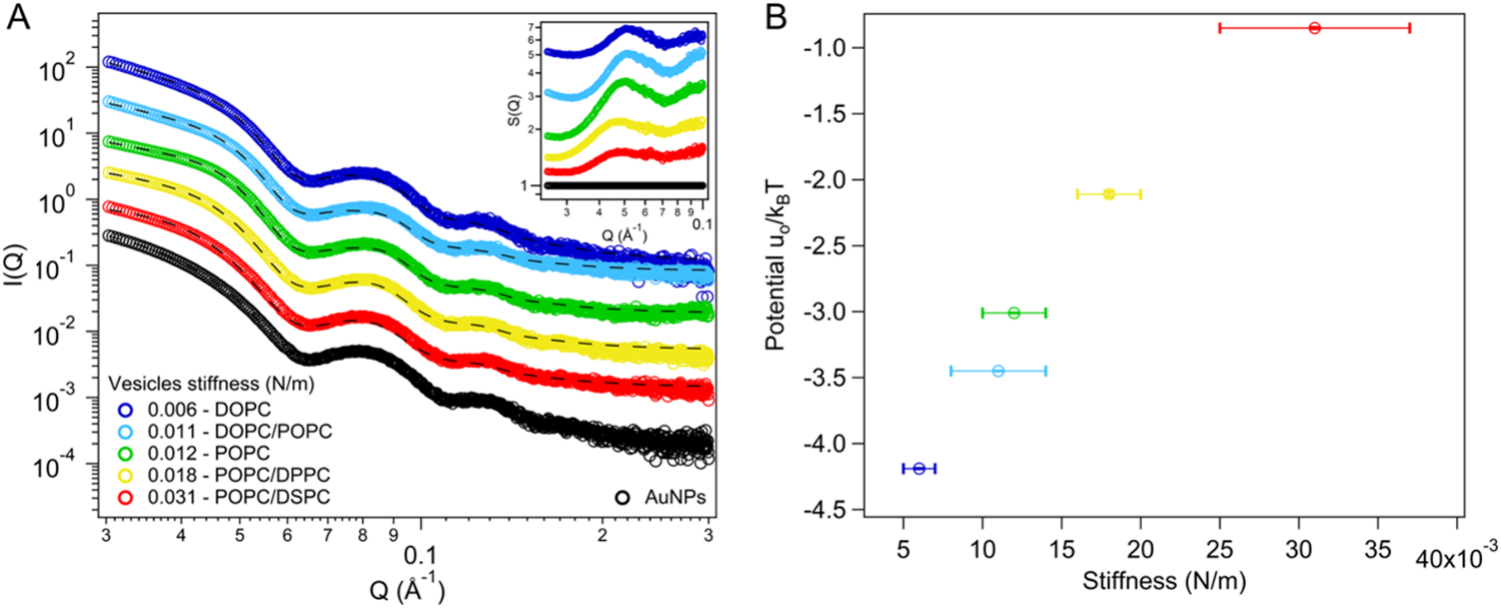
(A) Log–log SAXS profiles of AuNPs (4.4·10^12^ particles/mL) interacting with vesicles (3.2·10^11^ particles/mL) of varying stiffness, collected after 5 min of incubation at room temperature. SAXS profiles were fitted (black dashed lines) through a Sticky Hard Sphere model. The right inset is the structure factor *S*(*Q*) vs *Q* for each hybrid, extracted from SAXS profiles. The profiles are shifted for the sake of clarity. (B) Effective pair potential *u_0_*/*k*_B_ *T* as a function of vesicles stiffness obtained from AFM-FS measurements.

**Table 3:** Structural Parameters for Vesicles-AuNP Assemblies Obtained from Fitting According to the Sticky Hard Sphere Model, Including the Volume Fraction (φ), Stickiness Parameter (τ), and Effective Pair Potential, Normalized by Thermal Energy (*u*_0_/*k*_B_ *T*)

sample	φ	τ	*u*_0_ (*k*_B_ *T*)
DOPC	0.0189 ± 0.0001	0.0634 ± 0.0001	–4.190 ± 0.002
DOPC/POPC	0.0531 ± 0.0001	0.0884 ± 0.0001	–3.450 ± 0.001
POPC	0.0946 ± 0.0001	0.1028 ± 0.0001	–3.010 ± 0.001
POPC/DPPC	0.1130 ± 0.0013	0.2017 ± 0.0031	–2.110 ± 0.020
POPC/DSPC	0.1515 ± 0.0001	0.5930 ± 0.0038	–0.851 ± 0.006

As the membrane stiffness increases, the volume fraction of AuNP clusters increases, while the stickiness parameter decreases. The effective pair potential between AuNPs clustered on the liposomal surface was determined from τ (see Section S3.3 for further details) and plotted as a function of the vesicle stiffness, as shown in Figure 6B. Notably, the computed AuNP–AuNP effective pair interaction energy gradually becomes more attractive as the softness of vesicles increases. This behavior aligns closely with an interaction mechanism recently proposed.^20,22^ According to this model, AuNP clustering proceeds through membrane adhesion, driven by Van der Waals attractive forces. This is followed by the AuNP wrapping by the lipid membrane, also driven by Van der Waals forces, which induces the release of citrate anions in the water phase following ligand exchange with membrane phospholipids. This leads to AuNP clustering due to electrostatic destabilization. A reduced rigidity of the lipid template allows AuNPs to penetrate deeper into the membrane, maximizing AuNP wrapping due to reduced membrane bending energy costs. The membrane wrapping extent, in turn, affects the release of citrate anions from the AuNP surface and thus the electrostatic repulsion between neighboring AuNPs, ultimately tuning the AuNP–AuNP strength of interaction and, consequently, the interparticle distance.

### 3.5 Nanoplasmonic Isosbestics to Quantify the Mechanical Properties of Extracellular Vesicles

Nanoplasmonic isosbestics might offer potential for analytical applications, e.g., quantification of the stiffness of biological nanosized vesicles. This mechanical property plays a crucial role in modulating cellular uptake, tumor accumulation, and endosomal and/or lysosomal escape as well as vesicle’s response to external stimuli.^63,64^ Additionally, in natural vesicles like EVs, variations in the mechanical properties serve as biomarkers for malignant conditions of parental cells.^65–68^ Despite its biological relevance, measuring membrane rigidity remains challenging, often requiring sophisticated instruments and specialized expertise.^32^ In this context, AuNP nanoplasmonics has recently emerged as a promising alternative able to quantify the mechanical properties of vesicles through a simple colorimetric assay.^31^ However, a key limitation of this approach is that AuNP plasmonic is sensitive to both stiffness (Figure 7A) and concentration (Figure 7C) of vesicles. This dependence is typically quantified using optical descriptors such as the aggregation index (AI),^21,32^ which assumes increasingly high values with enhancing AuNP clustering.

**Figure 7:**
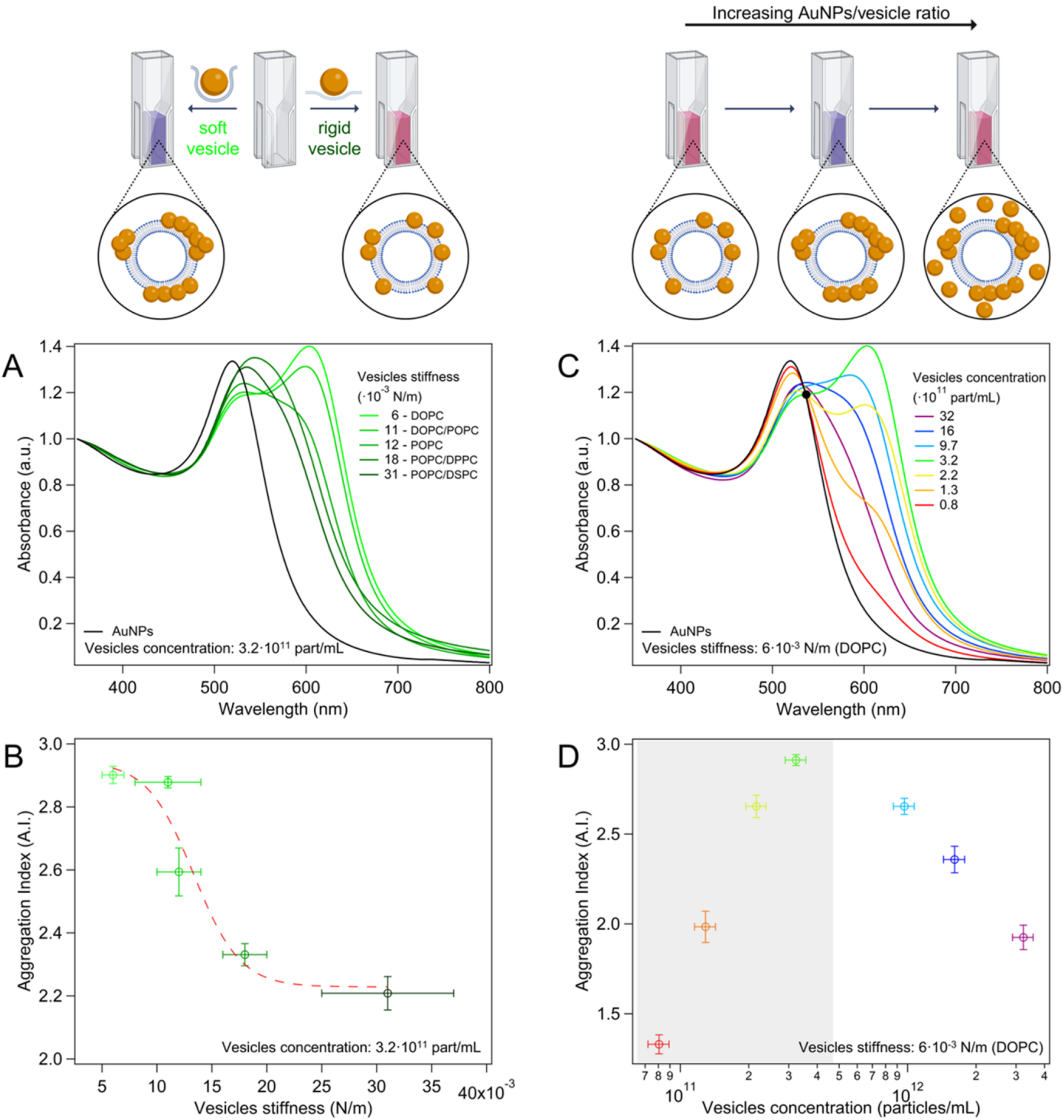
UV–vis spectra of AuNPs (4.4·10^12^ particles/mL) incubated with liposomal formulations of varying stiffness at a fixed concentration (3.2·10^11^ particles/mL) (A), and corresponding AI variations as a function of liposome stiffnesses (B); UV–vis spectra of AuNPs (4.4·10^12^ particles/mL) incubated DOPC liposomes at different concentrations (8·10^10^–3.2·10^12^ particles/mL) (C), and corresponding AI variations vesicle concentration (D). The black dot in panel (C) marks the isosbestic point where spectra recorded at low vesicle concentrations intersect. This feature is lost at higher vesicle concentrations, where the spectra no longer cross at this wavelength. The vesicle concentration range in which the isosbestic point emerges is highlighted in gray in panel (D). Error bars represent the standard deviation of independent triplicates. Elements of this figure were created in BioRender. Zendrini, A. (2025) (https://BioRender.com/308kucw).

As shown in Figure 7B, the AI decreases with increasing vesicle stiffness, following a sigmoidal trend (see Tables S6 and S7 for AI values with standard deviations and sigmoidal fitting parameters, respectively). In contrast, the AI exhibits a nonmonotonic dependence on vesicles concentration (Figure 7C,D and Table S8 for AI values with standard deviations). In the low vesicle concentration regime, AuNPs are in large excess compared to vesicles (from ∼74/1 to ∼14/1 AuNPs/liposomes number ratios), and AuNP aggregation increases with vesicles concentration. This corresponds to the region where the isosbestic point emerges (see the black dot in Figure 7C, indicating the isosbestic point where UV–vis spectra acquired at low vesicle concentrations intersect, and the corresponding vesicle concentration range highlighted in gray in Figure 7D). Given the large excess of AuNPs in this concentration regime, the membrane area is not sufficient to accommodate the whole AuNP population. As a result, vesicles become saturated with AuNPs, leading to the coexistence of free AuNPs in solution, alongside AuNP clusters. The emergence of the isosbestic point indicates that such clusters have uniform structural features, in terms of interparticle spacing (modulated by the rigidity of the template), but also of the cluster size. This can be explained by considering that under vesicle saturation conditions, AuNP clusters likely adopt the largest possible size allowed by electrostatic and vesicle size-related constraints. As the vesicle concentration increases within this regime, additional liposomes become available for aggregation and become saturated with AuNPs. This results in a higher number of clusters, as is evident from the steady increase in the AI, while the cluster size remains unchanged.

In contrast, in the high vesicle concentration regime (where AuNP are no longer in excess), increases in the vesicle/AuNP ratio decrease the AI. Additionally, in this regime, the isosbestic feature is lost (see Figure 7C where the spectra at high liposome concentrations no longer cross at the isosbestic wavelength). This is likely due to cluster size heterogeneity developing below vesicle saturation, where the available membrane surface is enough to accommodate all AuNPs and more, placing no constraints on the cluster size. Under these conditions, the free AuNP population disappears and clusters likely form with variable (and generally smaller) sizes, reducing the AI and resulting in the loss of the isosbestic feature. This concentration-dependent trend was consistent across all of the vesicle compositions investigated (see Figure S8 and Table S9).

The bimodal dependence of AuNP LSPR on vesicle stiffness and concentration poses a challenge for analytical applications as the accurate determination of one parameter (e.g., stiffness) requires prior knowledge of the other (e.g., concentration).

Conversely, our findings reveal that nanoplasmonic isosbestics—wavelengths that remain unchanged regardless of vesicle concentration—are governed only by membrane stiffness. Thus, λ_iso_ can serve as a robust, concentration-independent metric for precise quantification of membrane rigidity.

To validate this approach, we analyzed biological samples of EVs derived from red blood cells (RBC-EVs) and mesenchymal stem cells (MSC-EVs) (see Section S5 for details on production and characterization). The stiffness of such EVs was first determined through an independent technique. Since single-particle nanoindentation experiments are often technically more challenging when performed on EVs instead of liposomes, the mechanical properties of EVs were assessed through AFM quantitative morphometry as described elsewhere.^46^ Briefly, the extent of an EV’s deformation upon adsorption on an electrostatically charged substrate can be quantified via its contact angle, which is linearly correlated to its stiffness (i.e., the lower the contact angle, the lower the vesicle’s stiffness).

Table S15 reports the average contact angle measured on all of the synthetic liposomes and natural EVs used in this study. At least 100 individual vesicles were measured for each sample.

Figure 8A,B shows UV–vis spectra of AuNPs incubated with RBC and MSC-EVs, following the protocol previously employed for liposomes (described in Section 2.7). Remarkably, isosbestic points are present for both samples, with λ_iso_ = 531 ± 1 nm for RBC-EVs and 529 ± 1 nm for MSC-EVs. These experimental isosbestic wavelengths directly provide the average interparticle spacing of AuNP clusters formed on EVs through the sigmoidal law of Figure 4C, yielding <sp> values of 0.7 ± 0.07 nm for RBC-EVs and 0.6 ± 0.07 nm for MSC-EVs.

**Figure 8:**
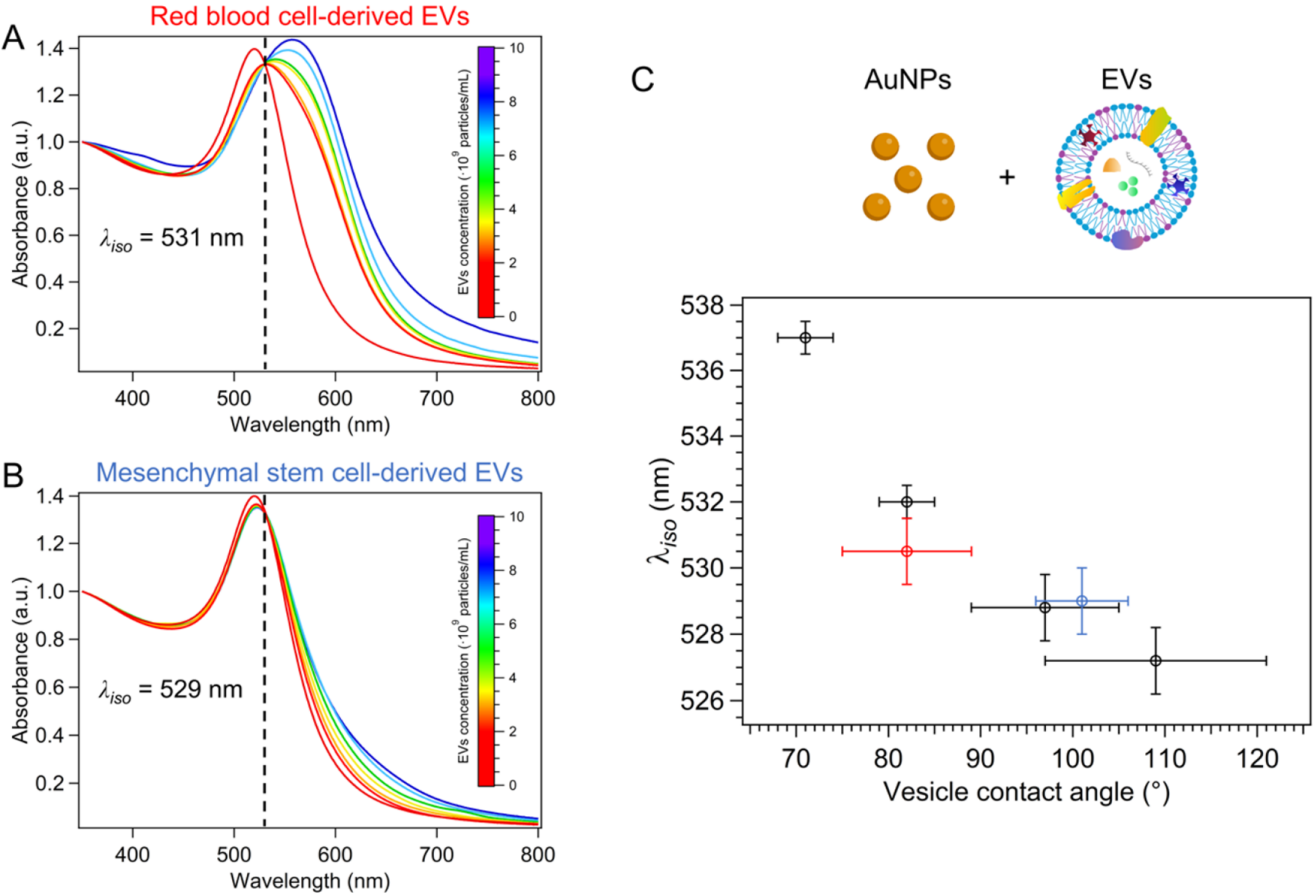
UV–vis spectra of AuNPs (4.4·10^12^ particles/mL) incubated with different concentrations of (A) RBC-EVs and (B) MSC-EVs, within the range of 2·10^9^–8·10^9^ particles/mL; (C) λ_iso_ variations as a function of the vesicle contact angle, with black dots referred to liposomes, and red and blue points representing RBC-EVs and MSC-EVs, respectively. Error bars represent the standard deviation of independent triplicates.

Additionally, these isosbestic wavelength values align with the AFM analysis (Figure 8C), indicating a higher rigidity for RBC-EVs compared with MSC-EVs. The results highlight the applicability of isosbestic points for quantifying the membrane stiffness, even in highly heterogeneous natural membranes. Notably, this method effectively operates at very low particle concentrations, which is convenient for biological samples, typically available in limited amounts.

## 4 Conclusions

Driven by their unique optical properties, plasmonic NP assemblies have encouraged significant research, stimulating advancements in medicine, biosensing, nanotechnology, and materials science. However, establishing a precise link between the nanostructure of NP assemblies and their optical properties remains a critical challenge.

In this study, we introduce nanoplasmonic isosbestics as a novel conceptual tool to unravel the relationship between AuNP assembly on soft templates and their plasmonic behavior. We used lipid vesicles to template the aggregation of AuNPs and demonstrate the occurrence of nanoplasmonic isosbestics over a specific range of AuNP-to-vesicle number ratios. Unlike previous works, where nanoplasmonic isosbestic points were observed during dynamic transformations between plasmonic species,^40,41^ we generated plasmonic isosbestic points under static conditions—i.e., in systems with a stable structural configuration. Numerical simulations indicated that these arise from the coexistence of two plasmonic species—free AuNPs and AuNP clusters—whose relative abundance is systematically varied by adjusting the molar ratio between liposomes and AuNPs, which in turn modulates the templating surface available for AuNP clustering. Additionally, we demonstrated that different interparticle spacings in AuNP clusters generate distinctive isosbestic wavelengths, showing for the first time that nanoplasmonic isosbestics can serve as unique optical fingerprints for structural features of AuNP assemblies on a specific soft template. For vesicle templates, <sp> gradually varies with membrane stiffness. In turn, membrane stiffness can be precisely tracked through shifts in the isosbestic wavelength, enabling the development of colorimetric assays for quantifying membrane rigidity in samples of unknown concentration.

Our findings establish nanoplasmonic isosbestics as powerful indicators of interparticle spacing and equilibrium structure in plasmonic assemblies. This approach offers a straightforward method to probe structure–function relationships in plasmonic systems and monitor the aggregation behavior of colloidal nanoparticles, thereby supporting the rational design of hybrid systems with controlled and customizable plasmonic properties. In this regard, the strategy holds significant translational potential, in terms of both material composition and scalability to larger plasmonic assemblies. Specifically, as citrate-stabilized AuNPs spontaneously cluster on a variety of synthetic (e.g., lipidic and polymeric)^20–30^ and natural (e.g., EVs and lipoproteins)^31–34^ soft matter materials, isosbestic monitoring can be adapted to track plasmonic colloidal assembly across a wide range of templates. Furthermore, larger patterns of AuNP clusters can be generated by using extended template areas for AuNP aggregation, such as cell-sized lipid or polymeric large unilamellar vesicles,^69^ or solid-supported bilayers^22,45^ and lipid films^70^ with lateral extension scalable up to the macroscale.

Finally, this approach lays the foundation for innovative applications in nanotechnology and plasmonic sensing, including detection of key properties of lipid vesicles of synthetic or natural origin and, more generally, of soft templates of biological relevance.

## Supplementary Material



## Notes

Evaluation of liposome concentration; DLS, ζ-potential, and SAXS results for liposomes and AuNPs; evaluation of the size and concentration of AuNPs from the analysis of UV–vis spectra; Cryo-EM imaging and SAXS data analysis of liposomes/AuNP samples; additional numeric simulations; production and characterization of EVs in terms of protein determination, and physicochemical characterization through DLS, ζ-potential measurements, NTA, and AFM imaging (PDF)

## ⋈

NEST, CNR Istituto Nanoscienze and Scuola Normale Superiore, Piazza San Silvestro 12, Pisa 56127, Italy
