# Supplementary material for: Nanoplasmonic Isosbestics Uncover Mesoscale Assembly of Gold Nanoparticles on Soft Templates
Source: J Am Chem Soc. 2025 May 30;147(23):20008–22. doi: 10.1021/jacs.5c05189 (PMC12164355; doi:10.1021/jacs.5c05189)
Supplement: Supplementary file 1 [file ja5c05189_si_001.pdf]

Nanoplasmonic Isosbestics Uncover Mesoscale Assembly of Gold Nanoparticles on Soft Templates

Jacopo Cardellini[1,2#], Ilaria De Santis[1,2#], Giuseppe Emanuele Lio[3,4], Marco Brucale[5], Francesco Valle[5], Virginia Catani[6], Ilenia Mastrolia[6], Marta Calabria[6], Massimo Dominici[6,7], Andrea Zendrini[2,8], Annalisa Radeghieri[2,8], Lucia Paolini[2,9], Paolo Bergese[2,8], Lucrezia Caselli[1,2*], Debora Berti[1,2] and Costanza Montis[1,2]

[#] *Equally contributed to this work*

Affiliations:

[1]Department of Chemistry "Ugo Schiff", University of Florence, 50019 Sesto Fiorentino, Florence, Italy

[2]CSGI, Center for Colloid and Surface Science, 50019 Florence, Italy

[3]European Laboratory for Non-Linear Spectroscopy (LENS), 50019 Sesto Fiorentino, Florence, Italy

[4]Department of Physics, University of Florence, 50019, Sesto Fiorentino, Florence, Italy.

[5]Istituto per lo Studio dei Materiali Nanostrutturati, CNR, 40129 Bologna, Italy

[6]Laboratory of Cellular Therapy, Department of Medical and Surgical Sciences for Children and Adults, University Hospital of Modena, Modena 41124, Italy

[7]Division of Medical Oncology, Department of Medical and Surgical Sciences for Children and Adults, University Hospital of Modena, Modena 41124, Italy

[8]Department of Molecular and Translational Medicine, University of Brescia, 25123 Brescia, Italy.

[9]Department of Medical and Surgical Specialties, Radiological Sciences and Public Health, University of Brescia, 25123 Brescia, Italy.

*Corresponding author:

Lucrezia Caselli

lucrezia.caselli@unifi.it

Keywords: plasmonics, gold nanoparticles, isosbestic points, Extracellular Vesicles, lipid membrane

S1 - Supplementary Characterization of Liposomes	**S3**
S1.1 - Evaluation of Liposomes Concentration	S3
S1.2 - Dynamic Light Scattering and ζ-Potential	S3
S1.3 - AFM Mechanical Characterization	S5
S1.4 - Small Angle X-ray Scattering	S5
S2 - Supplementary Characterization of Gold Nanoparticles	**S6**
S2.1 - Small Angle X-ray Scattering	S6
S2.2 - Dynamic Light Scattering and ζ-Potential	S6
S2.3 - UV-Vis Spectroscopy	S7
S3 - Supplementary Characterization of Vesicles-AuNPs assemblies	**S9**
S3.1 - Cryo-TEM	S9
S3.2 - UV-Vis Spectroscopy	S11
S3.3 - Small-Angle X-Ray Scattering	S15
S4 - Supplementary Numerical Simulations	**S19**
S4.1 - Numerical Scattering and Extinction cross sections	S19
S4.2 - Numerical Simulations varying the AuNPs radius (r = 12 nm)	S20
S5 - Supplementary Characterization of Extracellular Vesicles	**S22**

S5.1 - Red blood cell-derived extracellular vesicles (RBC-EVs) and mesenchymal stem cell-derived extracellular vesicles (MSC-EVs) production	S22
S5.2 – Dynamic Light Scattering and ζ-Potential	S25
S5.3 - Nanoparticle tracking analysis and protein content analysis	S25
S5.4 - Atomic Force Microscopy (AFM)	S26
References	S27

S1 - Supplementary Characterization of Liposomes

S1.1 - Evaluation of Liposomes Concentration

The lipid concentration in the starting colloidal dispersion was estimated to be 4 mg/mL from the initial lipid and water amounts employed in the formation and swelling of lipid films, assuming the absence of lipid loss due to the extrusion procedure. The liposomes concentration in the final dispersion was subsequently calculated considering the hydrodynamic diameter of each liposomal batch. In particular, the liposomal surface area (surface area = $4\pi r^2$) can be extracted from the liposome diameters; the doubled surface can be subsequently divided by the lipid cross section (approximately 0.5 nm^2) in order to obtain the lipid number per liposome, assuming that approximately one half of the lipids is localized in the external leaflet of a liposome, since the bilayer thickness, about 4-5 nm, is negligible with respect to the liposomes' average diameter. Eventually, the total weighted lipid concentration was divided by the total number of lipids per liposome, yielding the liposome concentration, which is reported in **Table S1** for each liposome dispersion.

Table S1. Final liposome concentration in each liposomal batch.

Sample	Liposomes Concentration (mol/L)	Liposomes Concentration (particles/mL)
DOPC	$3.8 \cdot 10^{-8}$	$2.3 \cdot 10^{13}$
DOPC/POPC	$3.1 \cdot 10^{-8}$	$1.9 \cdot 10^{13}$
POPC	$3.7 \cdot 10^{-8}$	$2.2 \cdot 10^{13}$
POPC/DPPC	$3.7 \cdot 10^{-8}$	$2.2 \cdot 10^{13}$
POPC/DSPC	$2.3 \cdot 10^{-8}$	$1.4 \cdot 10^{13}$

S1.2 - Dynamic Light Scattering and ζ-Potential

The hydrodynamic size of liposome dispersions was investigated through Dynamic Light Scattering. These data can be obtained by fitting the autocorrelation function of the scattering intensity, $g(\tau)$, as a function of time (τ). The decay rate of $g(\tau)$ is strictly related to the size of the scattering objects (the smaller the size, the fastest the decay of the autocorrelation function. **Figure S1** reports the DLS curves normalized in the form of $g_2(q, \tau)$ vs τ (in microseconds), measured for each sample. Each curve was analysed with the CONTIN fitting, to obtain the hydrodynamic diameter (D_h) and polydispersity (PDI) reported in **Table S2**.

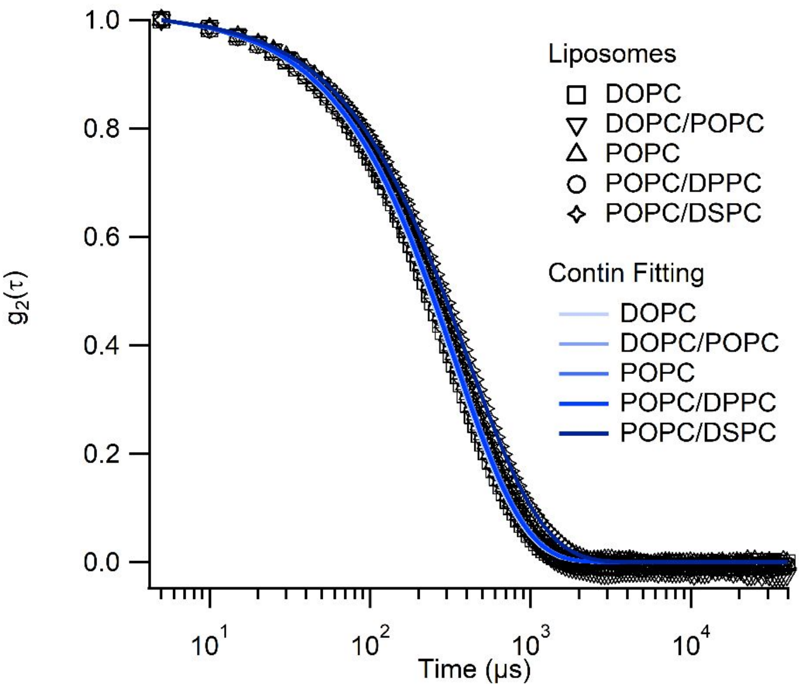

Figure S1. Autocorrelation functions of liposomes.

The surface charge of liposomes in Milli-Q water was evaluated through ζ-Potential measurements and is reported in **Table S2**.

Table S2. Hydrodynamic diameter and PDI obtained from Dynamic Light Scattering and ζ-Potential values of synthetic liposomes.

Sample	D_h (nm)	PDI	ζ-Potential (mV)
DOPC	103 ± 2	0.041 ± 0.025	0.2 ± 3.1
DOPC/POPC	116 ± 4	0.058 ± 0.039	-3.3 ± 3.4
POPC	106 ± 1	0.112 ± 0.040	-4.1 ± 1.8
POPC/DPPC	108 ± 1	0.033 ± 0.017	-4.5 ± 2.5
POPC/DSPC	134 ± 11	0.114 ± 0.032	-5.1 ± 1.0

S1.3 - AFM Mechanical Characterization

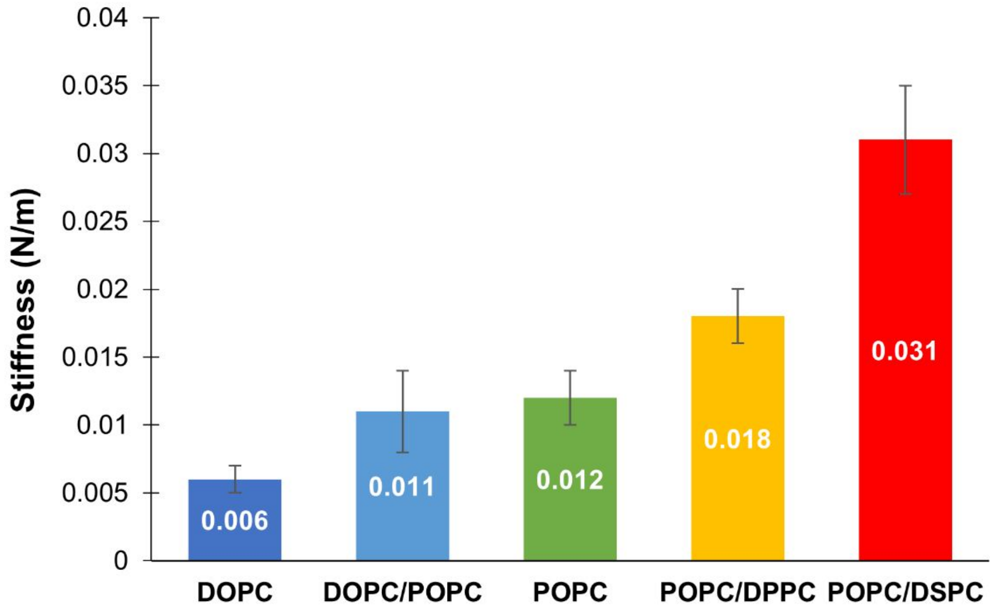

Figure S2. Stiffness values (N/m) of the different synthetic vesicles, determined through AFM-FS. All error bars represent the uncertainties obtained by bootstrapping (1000 repetitions of 5 draws, with replacement).

S1.4 - Small Angle X-ray Scattering

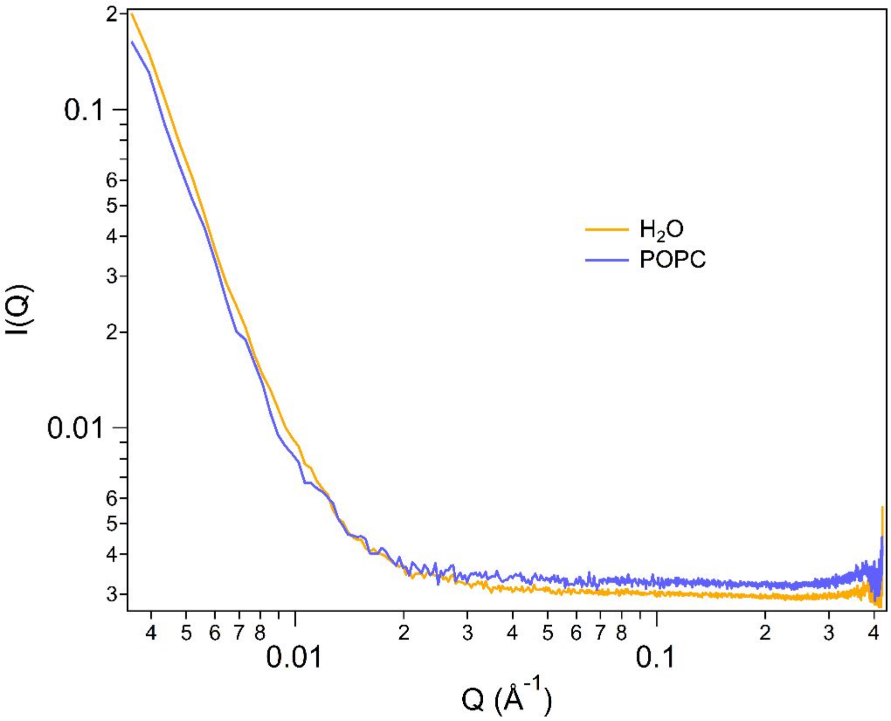

Figure S3. Comparison between the scattering intensity of the water medium and the scattering intensity of liposomes at a concentration of $3.2 \cdot 10^{11}$ particles/mL.

S2 - Supplementary Characterization of Gold Nanoparticles

S2.1 - Small Angle X-Ray Scattering

The structural parameters (**Table S3**) of citrate-coated gold nanoparticles were evaluated from their SAXS profile acquired at a concentration of $4.4 \cdot 10^{12}$ particles/mL and fitted according to a spherical form factor coupled to a Schulz size distribution[1] (**Figure S4**). The structural parameters obtained from fitting are reported in **Table S3**.

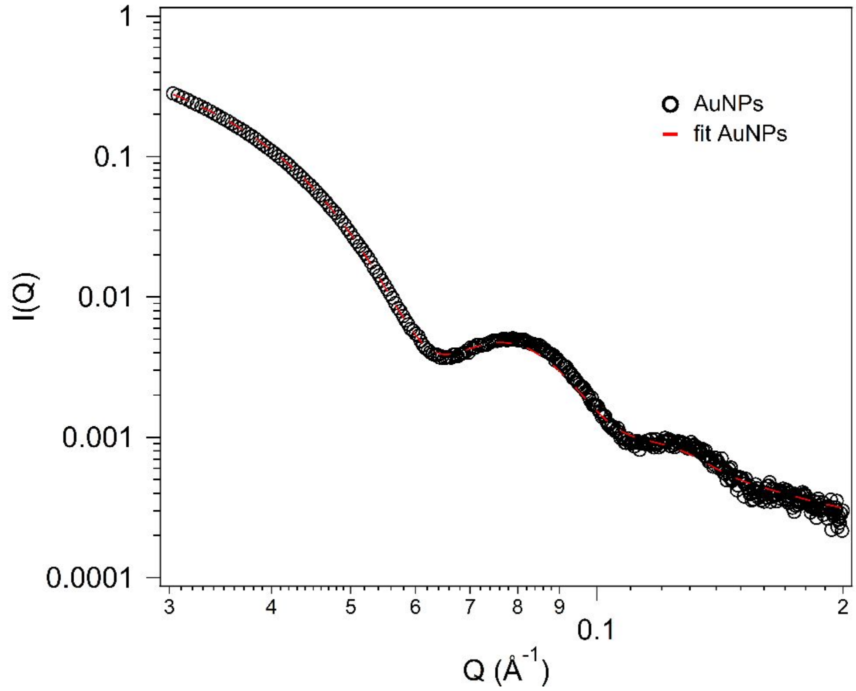

Figure S4. Experimental SAXS profile (black markers) and curve fit (dashed red line) according to the Schulz spheres model from the analysis software package SasView. The size and polydispersity obtained from the fitting procedure are summarized in the **Table S3** below.

Table S3. Structural parameters obtained from the analysis of SAXS profiles according to the Schulz spheres model, i.e., core radius (**R_{core}**) and polydispersity (PDI).

	**R_{core} (nm)**	**PDI**
AuNPs	6.86	0.130

S2.2 - Dynamic Light Scattering and *ζ*-Potential

AuNPs hydrodynamic diameter and surface charge in Milli-Q water were evaluated through Dynamic Light Scattering and ζ-Potential, respectively, and reported in **Table S4**.

Table S4. Hydrodynamic diameter (D_h) obtained from Dynamic Light Scattering and surface ζ-Potential values of AuNPs.

	D_h (nm)	ζ-Potential (mV)
AuNPs	20 ± 1	-35 ± 3

S2.3 - UV-Vis Spectroscopy

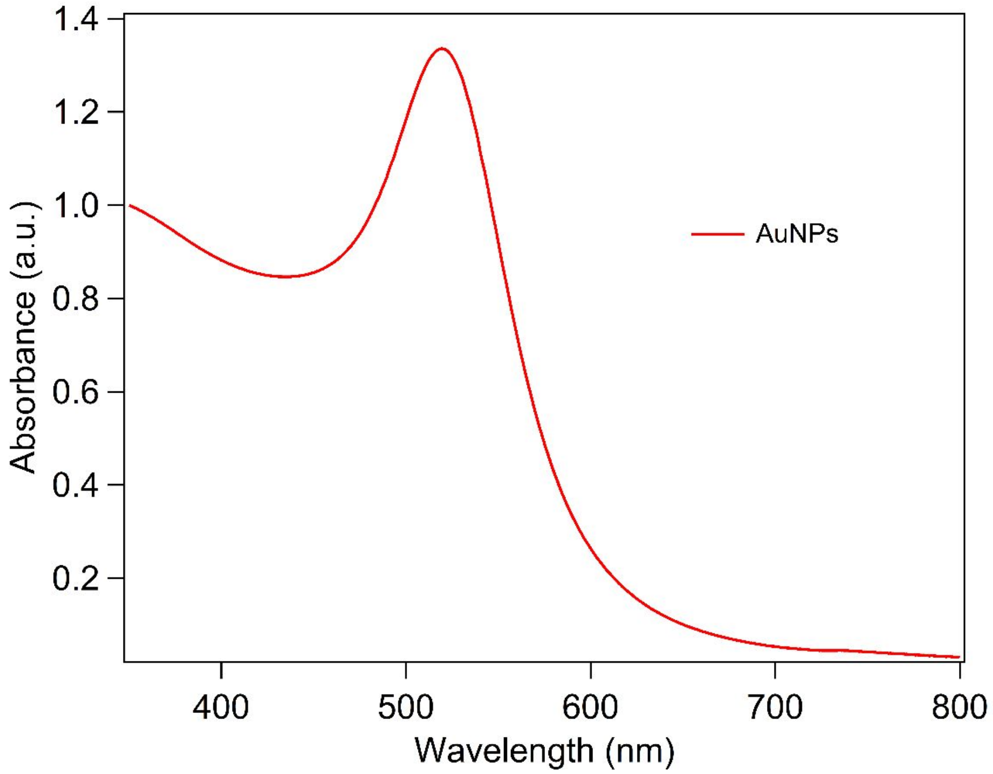

Figure S5. UV-Vis absorption spectra of AuNPs in MilliQ water at a concentration of $4.4 \cdot 10^{12}$ particles/mL.

To evaluate the AuNPs size from the UV-Vis spectrum of AuNPs of **Figure S5** we exploited the following equation[1]:

$$d = exp\left(B_1 \frac{A_{spr}}{A_{450}} - B_2\right)$$

with d diameter of gold nanoparticles, A_{spr} absorbance at the surface plasmon resonance peak, A_{450} absorbance at the wavelength of 450 nm and B_1 and B_2 dimensionless parameters, taken as 3 and 2.2, respectively. The diameter value obtained is 12.0 nm.

The concentration of citrate-coated gold nanoparticles was determined via UV-Vis spectroscopy, using the Lambert-Beer law (*E(λ) = ε(λ)lc*), taking the extinction values *E(λ)* at the LSPR maximum,

i.e. λ = 520 nm. The extinction coefficient *ε(λ)* of the AuNP dispersion was determined through a method reported in literature[2], by the following equation:

$$ln(\varepsilon) = k ln(d) + a$$

with d core diameter of nanoparticles (calculated as described previously), and k and a dimensionless parameters (k = 3.32111 and a = 10.80505)[3], leading to a *ε(λ)* of $1.8 \cdot 10^{8}$ $M^{-1} \cdot cm^{-1}$. The final concentration of the citrate-coated AuNPs is therefore ≈$7.5 \cdot 10^{-9}$ M (or $4.5 \cdot 10^{12}$ particles/mL).

S3 - Supplementary Characterization of Vesicles-AuNPs assemblies

S3.1 - Cryo-EM

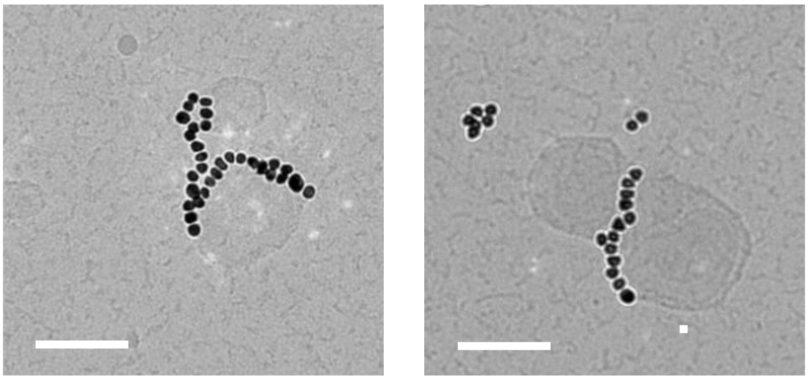

Figure S6. Representative cryo-EM images of AuNPs ($4.4 \cdot 10^{12}$ particles/mL) interacting with DOPC liposomes ($3.2 \cdot 10^{11}$ particles/mL) in MilliQ water. Scale bar 100 nm.

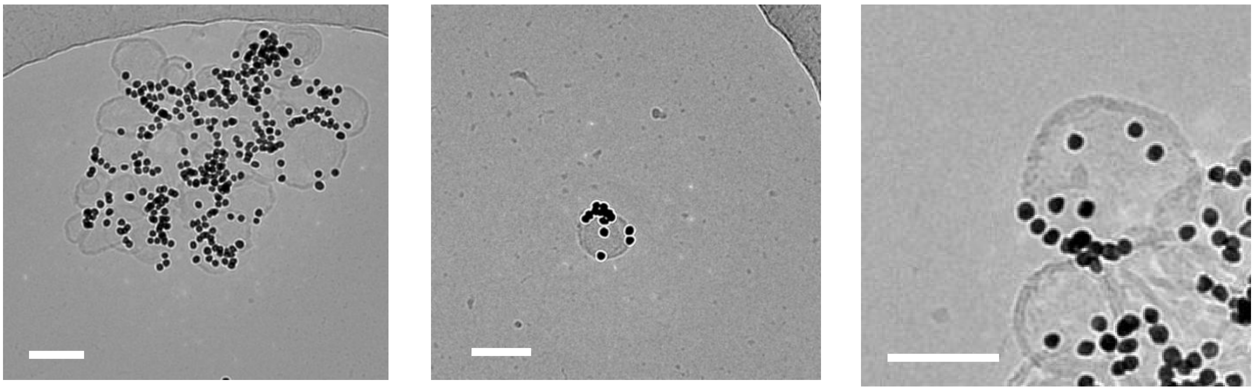

Figure S7. Representative cryo-EM images of AuNPs ($4.4 \cdot 10^{12}$ particles/mL) interacting with DOPC/DPPC liposomes ($3.2 \cdot 10^{11}$ particles/mL) in MilliQ water. Scale bar 100 nm.

S3.2 - UV-Vis Spectroscopy

The isosbestic points λ_{iso} for each liposomes/AuNPs assemblies (refer to **Figure 4** of the main text) are reported in **Table S5**, together with the relative standard deviation obtained from three repeated measurements on different samples.

Table S5. Mean isosbestic wavelengths and standard deviations for each liposome-AuNPs assemblies.

Sample	λ_{iso} (nm)
DOPC	537 ± 1
DOPC/POPC	536 ± 1
POPC	532 ± 1
POPC/DPPC	529 ± 1
POPC/DSPC	527 ± 1

The extent of AuNPs aggregation on synthetic liposomes can be described by using an aggregation index (A.I.), which is extracted dividing the area relative to the absorbance spectrum within the 560-800 nm range by the area subtended by the total spectral range (350-800 nm).[4] The calculated A.I. values for each hybrid system are then normalized for the one determined for the neat AuNPs.[4] The normalized A.I. value of isolated AuNPs is equal to 1; consequently, as the plasmonic variations become significant, indicating a higher degree of AuNPs aggregation, the A.I. increases accordingly.

The A.I. values for each liposomes/AuNPs assemblies of **Figure 6B** of the main text are reported in **Table S6**, together with the relative standard deviation obtained from three repeated measurements on different samples.

Table S6. A.I. mean values and standard deviations for each liposome-AuNPs hybrid, extracted from UV-Vis spectra of **Figure 7B**.

**Sample**	**A.I.**
DOPC	2.902 ± 0.027
DOPC/POPC	2.879 ± 0.018
POPC	2.594 ± 0.076
POPC/DPPC	2.331 ± 0.035
POPC/DSPC	2.208 ± 0.053

The fitting parameters describing the sigmoidal best fit for the A.I. values of AuNPs-liposomes systems plotted versus the stiffness determined through AFM-FS are the following:

Table S7. Fitting parameters obtained by fitting the A.I. vs stiffness values of **Figure 7B**, through the sigmoidal best fit.

**a**	**b**	**c**	**d**	χ^2
2.945 ± 0.209	-0.716 ± 0.286	0.013 ± 0.003	0.002 ± 0.002	0.025

The A.I. values obtained for DOPC-AuNPs systems by varying DOPC concentration (refer to **Figure 7D** of the main text) are reported in **Table S8**, together with the relative standard deviation obtained from three repeated measurements on the different samples.

Table S8. A.I. mean values and standard deviations for DOPC-AuNPs hybrids obtained by varying DOPC concentration, extracted from UV-Vis spectra of **Figure 7D**.

DOPC conc. (part/mL)	A.I.
$8.1 \cdot 10^{10}$	1.330 ± 0.053
$1.3 \cdot 10^{11}$	1.984 ± 0.087
$2.2 \cdot 10^{11}$	2.655 ± 0.062
$3.2 \cdot 10^{11}$	2.902 ± 0.027
$9.7 \cdot 10^{11}$	2.654 ± 0.045
$1.6 \cdot 10^{12}$	2.358 ± 0.074
$3.2 \cdot 10^{12}$	1.925 ± 0.068

Similar UV-Vis investigations were performed on DOPC/POPC-, POPC-, POPC/DPPC- and POPC/DSPC-AuNPs systems by varying vesicles concentration. The corresponding A.I. values plotted as a function of vesicles concentration are reported in **Figure S9** for each liposomes-AuNPs hybrid (see **Table S9** for A.I. values with relative standard deviations, obtained from three repeated measurements on the different samples).

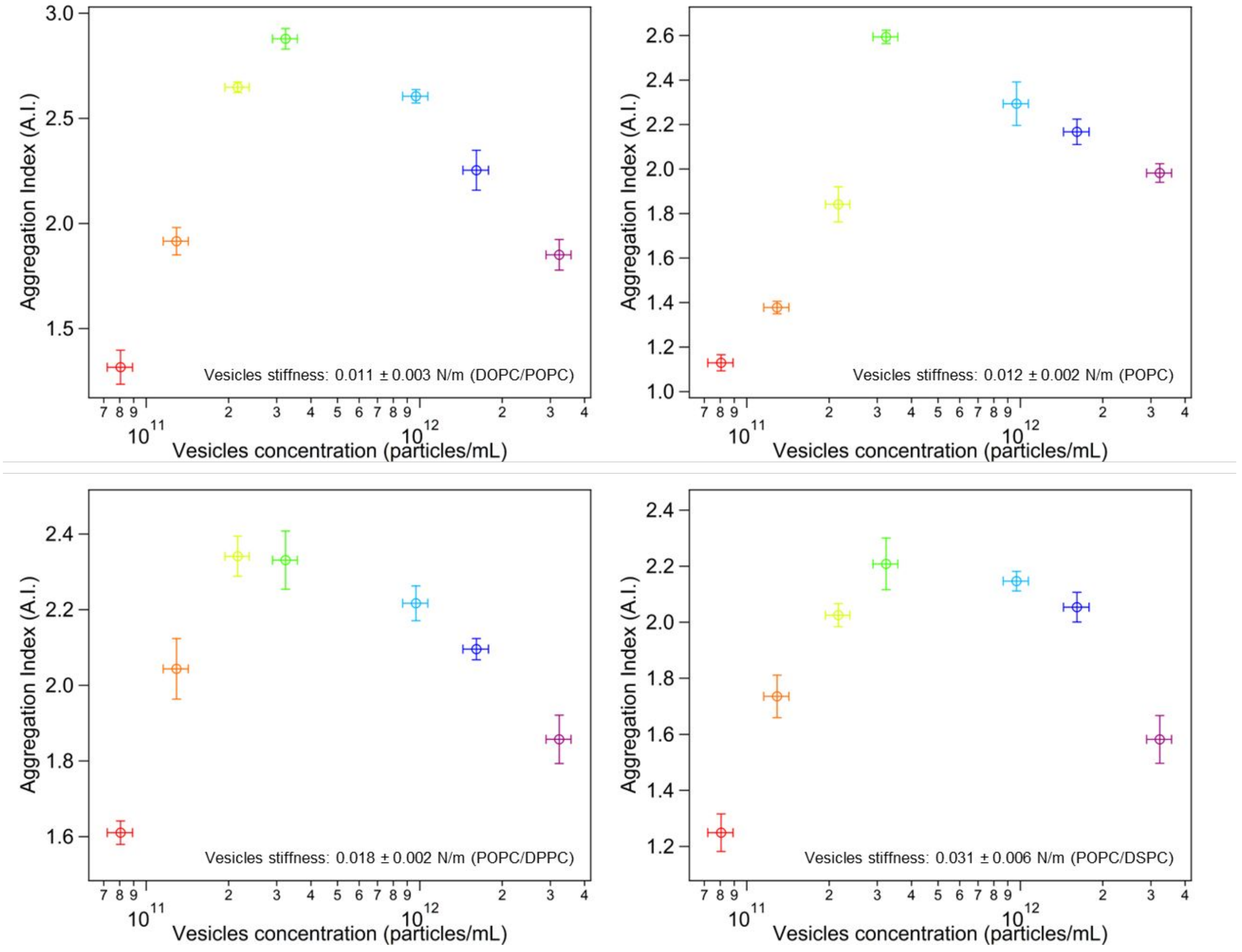

Figure S8. Corresponding A.I. values plotted as a function of vesicles concentration in the range $8.1 \cdot 10^{10}$ - $3.2 \cdot 10^{12}$ particles/mL.

Table S9. A.I. mean values and standard deviations for vesicles-AuNPs hybrids obtained by varying vesicles concentration.

**Vesicles conc. (part/mL)**	**A.I. DOPC/POPC**	**A.I. POPC**	**A.I. POPC/DPPC**	**A.I. POPC/DSPC**
$8.1 \cdot 10^{10}$	1.330 ± 0.053	1.129 ± 0.036	1.611 ± 0.031	1.249 ± 0.067
$1.3 \cdot 10^{11}$	1.984 ± 0.087	1.378 ± 0.028	2.043 ± 0.080	1.735 ± 0.076
$2.2 \cdot 10^{11}$	2.655 ± 0.062	1.841 ± 0.079	2.341 ± 0.053	2.025 ± 0.041
$3.2 \cdot 10^{11}$	2.902 ± 0.027	2.594 ± 0.030	2.331 ± 0.077	2.208 ± 0.092
$9.7 \cdot 10^{11}$	2.654 ± 0.045	2.294 ± 0.098	2.217 ± 0.046	2.147 ± 0.035
$1.6 \cdot 10^{12}$	2.358 ± 0.074	2.167 ± 0.057	2.096 ± 0.028	2.054 ± 0.053
$3.2 \cdot 10^{12}$	1.925 ± 0.068	1.982 ± 0.042	1.857 ± 0.064	1.582 ± 0.085

S3.3 - Small-Angle X-Ray Scattering

SAXS measurements on liposomes-AuNPs aqueous dispersion were recorded at the ID02 beamline at the European Synchrotron Radiation Facility (ESRF, Grenoble, France) and carried out in sealed glass capillaries of 1.5 mm diameter. SAXS profiles of liposomes-AuNPs in an extended Q range ($0.0035 \leq Q \leq 0.4218$ Å^{-1}) are reported in **Figure S10**. The power-law dependence in the low-*q* region, obtained through linear fitting of log-log SAXS profiles (see **Figure S11** and **Table S10**), highlights the presence of AuNPs clusters, with a fractal dimension which increases as the stiffness of vesicles decreases.

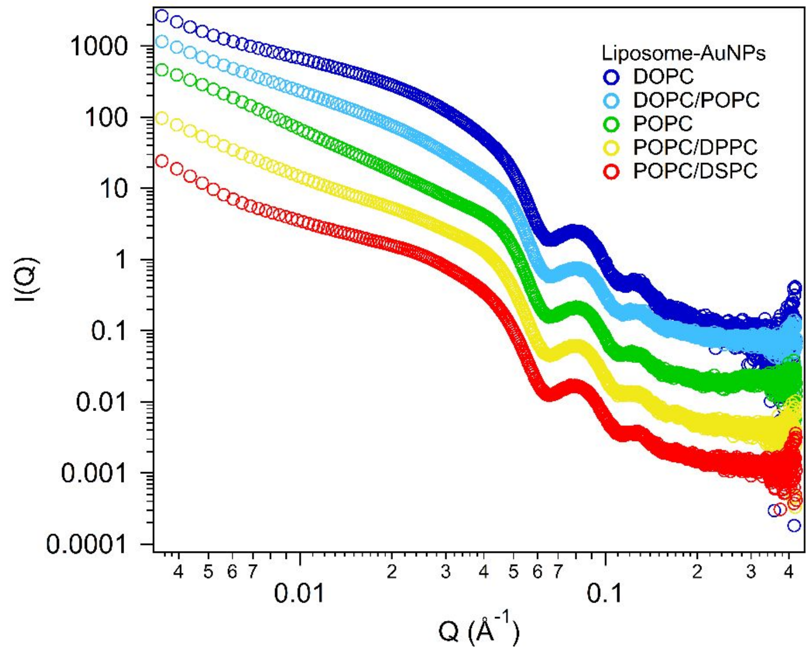

Figure S9. Experimental SAXS profiles obtained for liposomes-AuNPs hybrids.

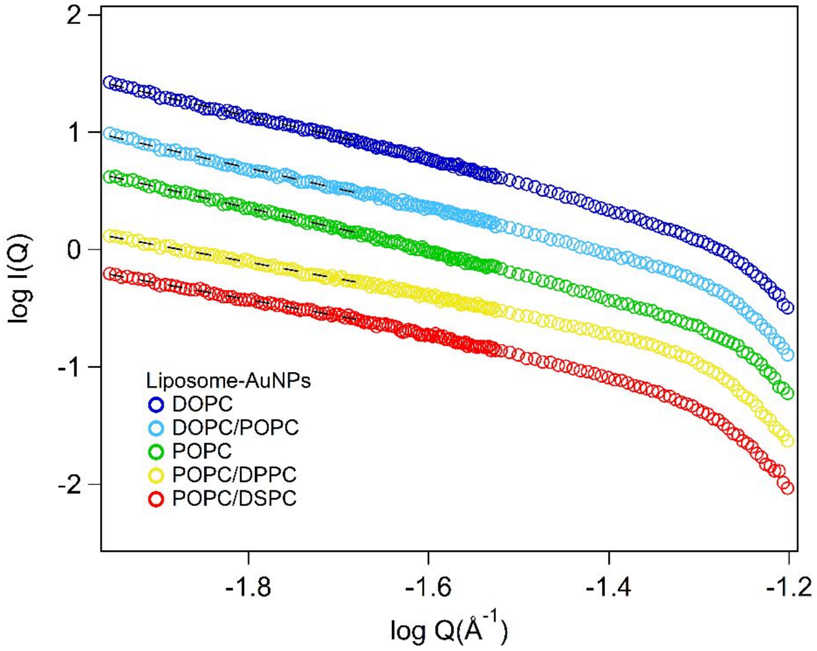

Figure S10. log-log SAXS profiles obtained for liposomes-AuNPs hybrids in the low Q region, together with linear fittings (dashed black lines) performed in the -1.9539 < log Q < -1.6714 range.

Table S10. Power-law exponents obtained from the linear fittings of log-log SAXS profile in the low-Q region.

Sample	Slope
DOPC	-1.771 ± 0.015
DOPC/POPC	-1.770 ± 0.020
POPC	-1.760 ± 0.014
POPC/DPPC	-1.422 ± 0.015
POPC/DSPC	-1.398 ± 0.014

The structure factors S(Q) for the scattering profiles reported in **Figure 5A** (main text) were obtained as follow. The scattering intensity I(Q) is defined by the equation:

$$I(Q) = KN_P V_P^2 (\Delta\rho)^2 P(Q) S(Q) + B$$

With K instrumental constant, N_P scattering nanoparticles' number per unit volume, V_P nanoparticle's volume, $\Delta\rho$ contrast of the experiment, B background intensity, $P(Q)$ and $S(Q)$ form and structure factors, respectively. To obtain S(Q) of liposome-AuNPs complexes ($S(Q)_{hyb}$), we divided the scattering intensity of the liposomes-AuNPs hybrids by the scattering intensity of the neat AuNPs dispersion:

$$\frac{I(Q)_{hyb}}{I(Q)_{AuNP}} \sim \frac{S(Q)_{hyb} P(Q)_{hyb}}{S(Q)_{AuNP} P(Q)_{AuNP}}$$

For a diluted AuNPs dispersion the structure factor can be considered equal to 1. In addition, in the high-Q region (0.03-0.3 $Å^{-1}$), the form factor of liposomes-AuNP hybrids can be approximated to the one of neat AuNPs, leading to the following equation:

$$\frac{I(Q)_{hyb}}{I(Q)_{AuNP}} = S(Q)_{hyb}$$

The mean center-to-center interparticle distance between AuNPs within the aggregates (d) can be obtained from the S(Q) vs Q ($Å^{-1}$) plot (see inset of **Figure 5A** of the main text), by the following equation:

$$d = \frac{2\pi}{Q_{max}}$$

With Q_{max} value corresponding to the maximum of the correlation peaks reported in the inset of **Figure 5A** (main text).

Table S11. Center-to-center interparticle distances calculated for the different AuNPs-liposomes hybrids.

Sample	Interparticle distance (nm)
DOPC	12.5
DOPC/POPC	12.5
POPC	12.6
POPC/DPPC	13.4
POPC/DSPC	13.4

The analysis of the structure factor S(Q) allows determination of the interparticle interaction potential. To this purpose, SAXS profiles 0.03-0.3 Å^{-1} Q-range (**Figure 5A**) were fitted combining a spherical form factor with a Schulz size distribution[5] with a Sticky Hard Sphere (SHS) interparticle structure factor[6,7], using the analysis software package SasView. The latter calculates the interparticle structure factor for a hard sphere fluid with a narrow, attractive, potential well.

The SHS potential consists of an attractive interaction between the particles, additional to a hard-core repulsion, and is defined as:

$$u(r) = \begin{cases} \infty & r < \sigma \\ -u_0 & \sigma \le r \le \sigma + \Delta \\ 0 & r > \sigma + \Delta \end{cases}$$

where r denotes the interparticle distance, σ is the hard particle diameter, Δ and u_0 are the width and depth of the square well, respectively.

The model contains the following fitting parameters:

- R_{eff}, defined as the effective radius of the hard sphere (described below);
- ϕ, which describes the volume fraction of the hard spheres;
- ε, which is a perturbation parameter (described below);
- τ, the stickiness parameter which describes the strength of the attractive well.

τ is defined in the equation below, as a function of both the perturbation parameter and the interaction in k_BT units:

$$\tau = \frac{1}{12\varepsilon} exp(\frac{u_0}{k_B T})$$

From the definition, smaller τ means a stronger attraction.

The perturbation parameter ε is defined as follows:

$$\varepsilon = \frac{\Delta}{\sigma + \Delta}$$

where σ is the hard sphere diameter and Δ is the mean interparticle distance <sp>. To fit SAXS profiles of **Figure 5A,** ε was calculated for each each hybrid sample, considering the diameter of individual AuNPs obtained from SAXS (see Section **S2**) and <sp> retrieved from numerical simulations (see section **3.2** of Results and discussion), and used as input parameter in the fitting.

The effective radius of the hard sphere R_{eff} is defined as follows:

$$R_{eff} = R + 0.5{\cdot}\Delta$$

where R is the AuNPs radius and Δ is the mean interparticle distance <sp>. Similarly to ε, R_{eff} was therefore calculated from the AuNP size obtained from SAXS and <sp> values obtained from numerical simulations and used as (fixed) input parameter in the fitting. Table S12 reports the values of ε, R_{eff} calculated as described above, and used as fitting parameters.

Table S12. Input parameters employed in the fitting according to the Sticky Hard Sphere model, i.e, effective hard sphere radius (R_{eff}) and the perturbation parameter (ε).

Sample	R_{eff} (nm)	ε
DOPC	7.01	0.02
DOPC/POPC	7.06	0.03
POPC	7.13	0.04
POPC/DPPC	7.21	0.05
POPC/DSPC	7.26	0.06

The fitting was done by fixing the X-ray scattering length density of H_2O and AuNPs at $9.47{\cdot}10^{-6}$ Å^{-2} and $125{\cdot}10^{-6}$ Å^{-2}, respectively.

The volume fraction of the aggregates ϕ and the stickiness parameter τ were left as free parameters.

Moreover, in the data fitting, we considered also the polydispersity effect due to the AuNPs polydispersity index equal to 0.130 (refer to the Section **S2**).

S4 - Supplementary Numerical Simulations

S4.1 - Numerical Absorbance and Scattering cross sections for AuNPs with 12 nm diameter

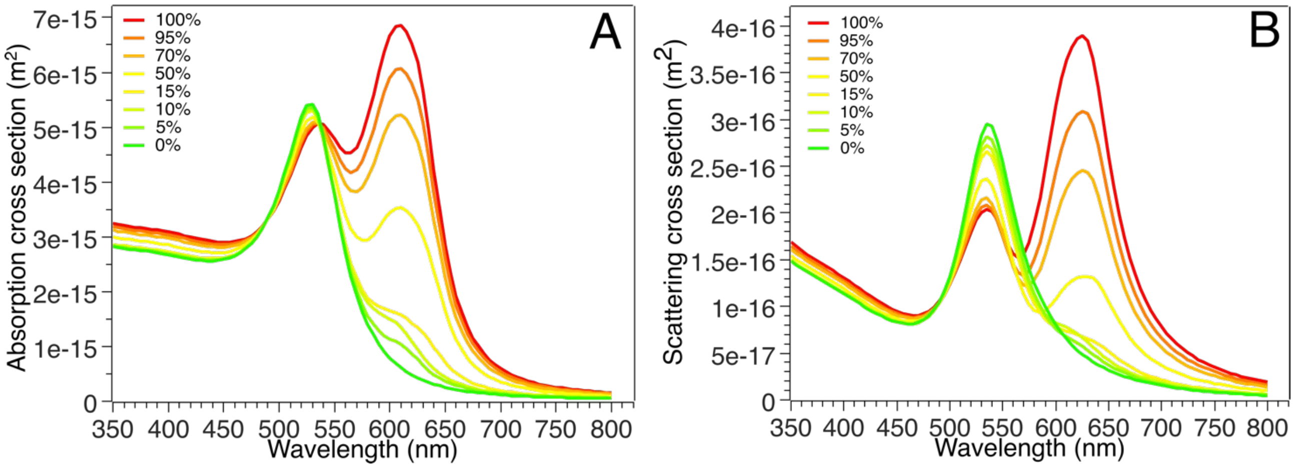

Figure S11. Numerical simulations displaying the absorption (A) and scattering (B) cross sections of AuNPs, obtained by varying the percentage of individual (uncoupled) AuNPs (<sp> = 4 nm) and plasmonically coupled AuNPs, with <sp> = 0.3 nm; in both plots the red solid line remarks the complete coupled condition (100% of plasmonically coupled of AuNPs), while the green solid line highlights the fully uncoupled condition (0% of plasmonically coupled AuNPs).

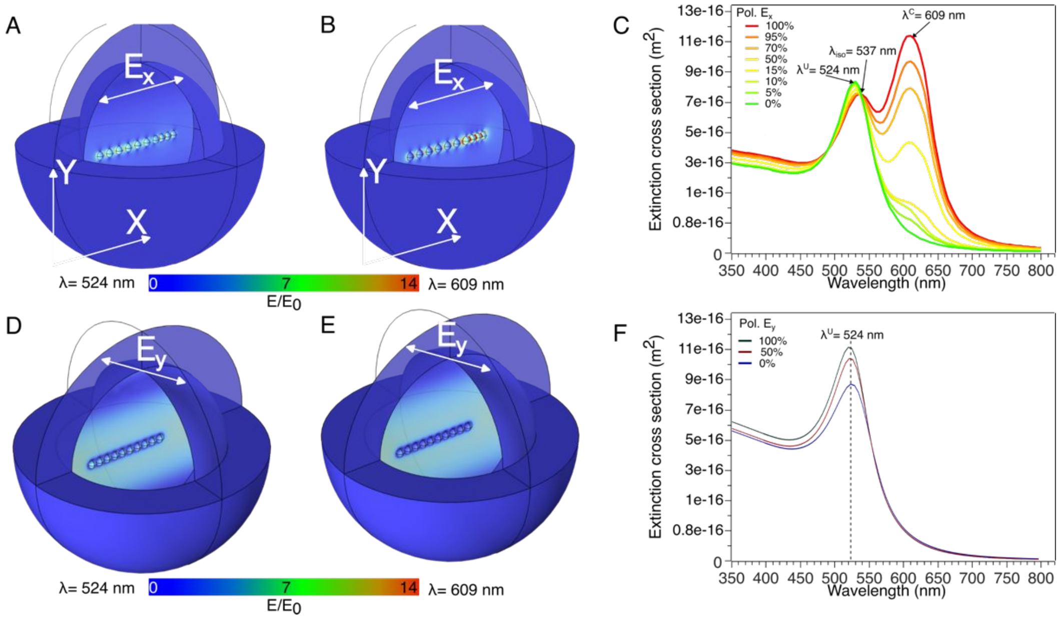

Figure S12. Numerical simulations of AuNPs arranged as a one-dimensional chain. Schematic illustrations of the configuration adopted using light polarized along the X direction and with wavelength λ=524 nm (A) and λ=609 nm (B), or light polarized along the Y direction, with wavelength λ=524 nm (D) and λ=609 nm (E). Corresponding extinction cross sections of AuNPs obtained by varying the percentage of individual (uncoupled) AuNPs (<sp>=4 nm) and plasmonically

coupled AuNPs (<sp> of 0.3 nm) are shown in (C) and (F), for light polarized along the X and Y directions, respectively.

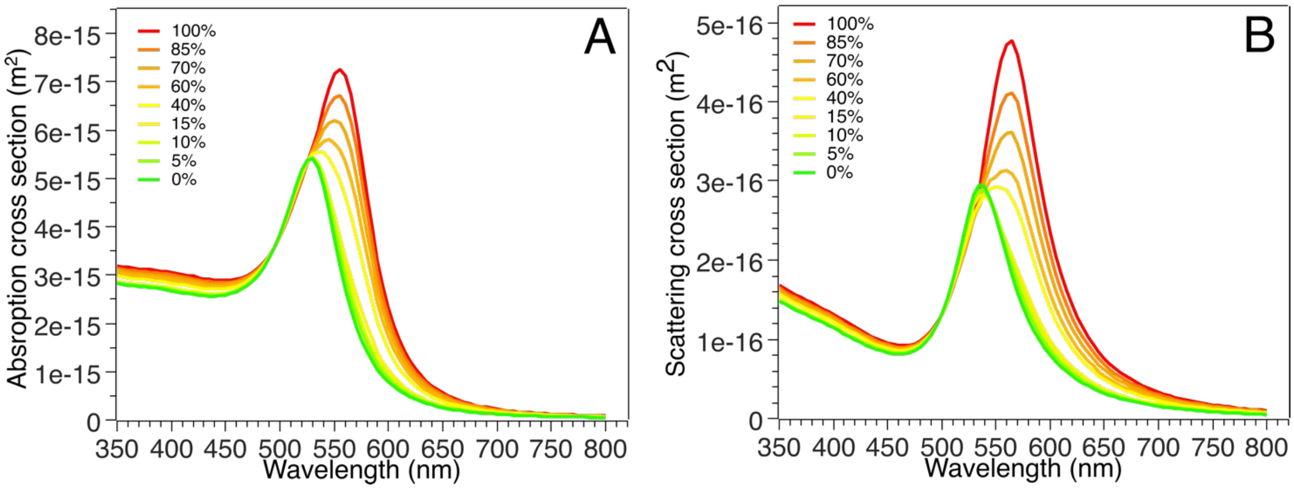

Figure S13. Numerical simulations displaying the absorption (A) and scattering (B) cross sections of AuNPs, obtained by varying the percentage of individual (uncoupled) AuNPs (<sp>=4 nm) and plasmonically coupled AuNPs, with <sp> = 0.8 nm; in both plots the red solid line remarks the complete coupled condition (100% of plasmonically coupled of AuNPs), while the green solid line highlights the fully uncoupled condition (0% of plasmonically coupled AuNPs).

S4.2 - Numerical simulations for AuNPs with 24 nm diameter

As a further test, numerical simulations have been performed for AuNPs of bigger size (diameter = 2 nm). To investigate the dependence of AuNP extinction cross section on <sp> within clusters, we systematically varied the spacing to cover a wide range of distances, from subnanometer to a few nanometers (**Figure S14**). At <sp> = 6 nm, AuNPs behave as individual plasmonic entities, showing a single plasmonic resonance close to the one expected for isolated nanoparticles. From <sp> = 1.2 nm to <sp> = 2.0 nm this resonance becomes increasingly broader, until a secondary plasmonic peak develop in the 0.6-1.0 nm <sp> interval.

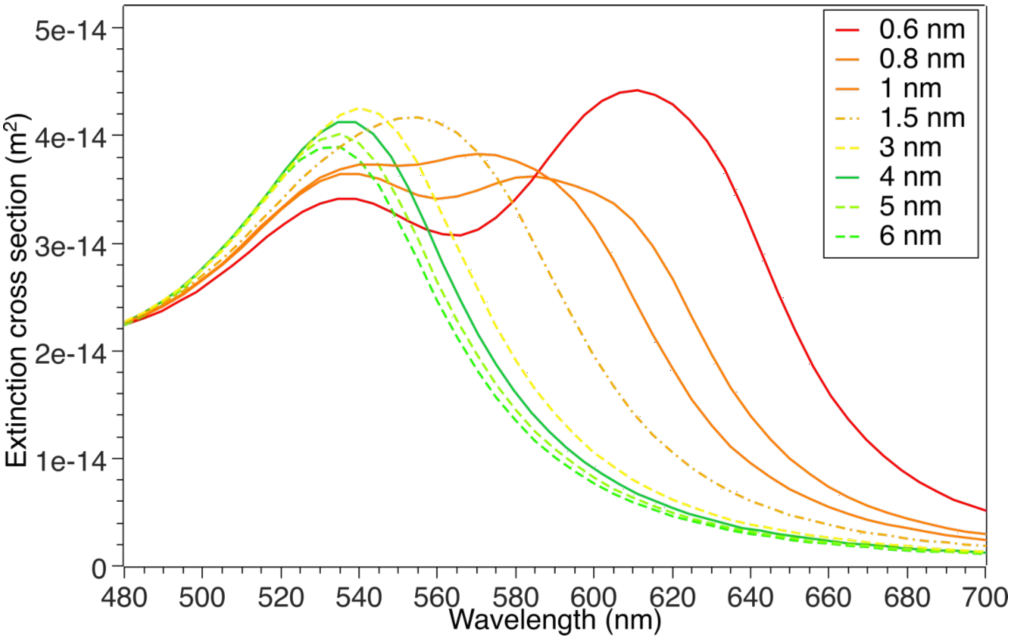

Figure S14. Extinction cross sections evaluated using numerical simulations considering a circular arrangement of AuNPs placed at different values of <sp>.

To assess the effect of varying the number of AuNPs involved in clustering, we analyzed a system comprising two populations (i.e., plasmonically coupled AuNPs and plasmonically uncoupled AuNPs) and systematically varied the percentages of such populations, while keeping the average <sp> within the coupled population constant at either 0.6 nm (**Figure S15**) or 1.5 nm (**Figure S16**). The <sp> of uncoupled AuNPs was kept constant at 4 nm. The simulated absorption, scattering and extinction cross sections for the two cases are reported below.

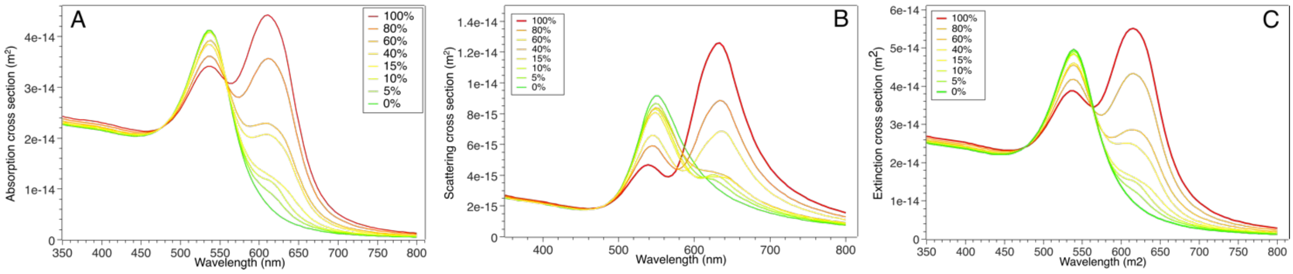

Figure S15. Numerical simulations displaying the absorption (A) and scattering (B) and extinction (C) cross sections of AuNPs, obtained by varying the percentage of individual (uncoupled) AuNPs (<sp> = 4 nm) and plasmonically coupled AuNPs, with <sp> = 0.6 nm; in both plots the red solid line remarks the complete coupled condition (100% of plasmonically coupled of AuNPs), while the green solid line highlights the fully uncoupled condition (0% of plasmonically coupled AuNPs).

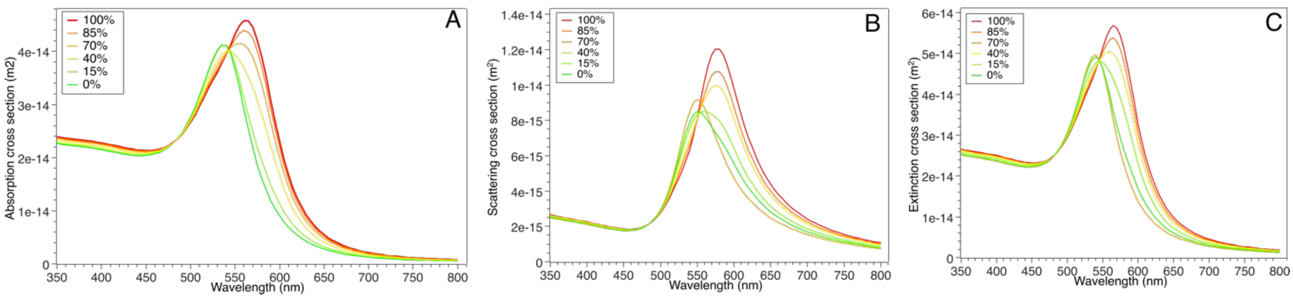

Figure S16. Numerical simulations displaying the absorption (A) and scattering (B) and extinction (C) cross sections of AuNPs, obtained by varying the percentage of individual (uncoupled) AuNPs (<sp> = 4 nm) and plasmonically coupled AuNPs, with <sp> = 1.5 nm; in both plots the red solid line remarks the complete coupled condition (100% of plasmonically coupled of AuNPs), while the green solid line highlights the fully uncoupled condition (0% of plasmonically coupled AuNPs).

Numerical simulations also in this case study allowed calculating the trend of the λ_{iso} in a wide range of <sp>, which can be fitted according to sigmoidal fit (see **Figure S17**).

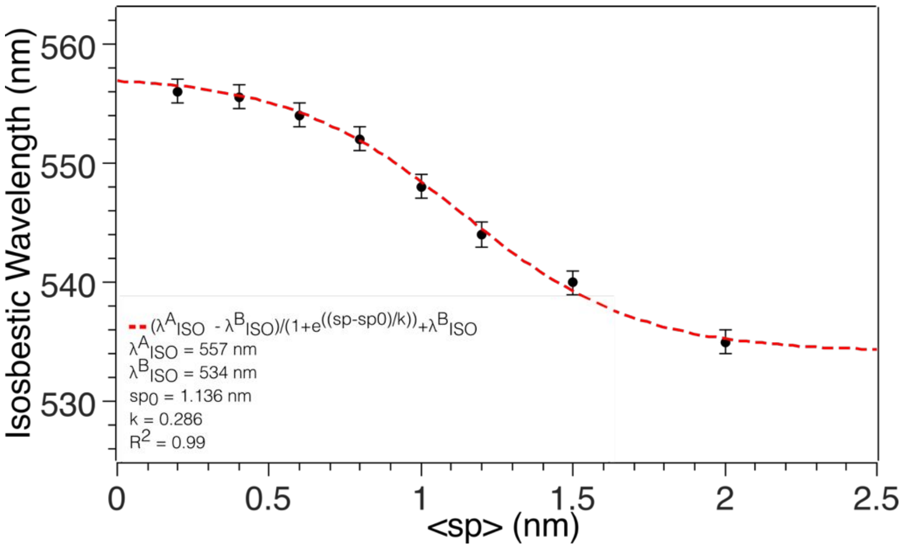

Figure S17. λ_{iso} values obtained from numerical simulations as function of <sp>, fitted according to a sigmoidal law described by the parameters indicated in the graph.

S5 - Supplementary Characterization of Extracellular Vesicles

S5.1 Red blood cell-derived extracellular vesicles (RBC-EVs) and mesenchymal stem cell-derived extracellular vesicles (MSC-EVs) production

Red blood cell-derived extracellular vesicles (RBC-EVs) were produced by chemical induction with $CaCl_2$ and calcium ionophore, as previously described by Usman et al.[8]. Briefly, 300 mL of RBC concentrates (sourced from anonymized healthy donors with written consent and supplied by the

Hospital Spedali Civili of Brescia, Brescia (Italy), ethical approval nr. NP5705) were centrifuged at 1,000 × g for 8 minutes at 4°C and washed twice with sterile PBS devoid of Ca^{2+} and Mg^{2+} (hereafter referred to as PBS). Each RBC pellet was then resuspended and washed with 50 mL of sterile PBS supplemented with 0.1 g/L of $CaCl_2$ (hereafter referred to as CPBS, in a 2:1 CPBS to RBC volume ratio), and centrifuged again at 1,000 × g for 8 minutes at 4°C. The washed RBC pellets were finally resuspended in 30 mL of fresh CPBS containing 10 µM calcium ionophore, transferred into 175 cm^2 tissue culture flasks (Sarstedt Ag & Co. KG, Nümbrecht, Germany), and incubated at 37°C (5% CO_2) for 15 hours.

After the overnight incubation, RBCs were diluted 1:1 with PBS, and RBC-EVs were isolated by multiple sequential centrifugation steps, all conducted at 4°C: 600 × g for 20 minutes, 1,600 × g for 15 minutes, 3,200 × g for 15 minutes, and 10,000 × g for 30 minutes. In each step, the supernatant was collected, and the pellets were discarded. The supernatant from the 10,000 × g centrifugation was then filtered using Primo syringe 0.45 µm PES filters (Euroclone S.p.A., Milan, Italy) and ultracentrifuged at 50,000 × g for 70 minutes at 4°C.

The RBC-EV pellet was resuspended in 2 mL of PBS and gently layered on top of 2 mL of ice-cold PBS, which had been deposited onto a 1 mL frozen 60% sucrose cushion in PBS. Samples were then ultracentrifuged at 50,000 × g for 16 hours at 4°C, with the deceleration speed set to 0. The red layers containing RBC-EVs were collected, resuspended, and washed in 1.5 mL of PBS each, then ultracentrifuged at 50,000 × g for 70 minutes at 4°C. Finally, each RBC-EV pellet was resuspended in 1 mL of PBS, diluted 10 times, aliquoted, characterized, and stored at –80°C until further use.

Every centrifugation below 10,000 × g was performed on a 5804R centrifuge equipped with an A-4-44 swinging bucket rotor (Eppendorf SE, Hamburg, Germany), using 50 mL sterile disposable Falcon tubes (Sarstedt Ag & Co, Nümbrecht, Germany). The 10,000 × g centrifugation step was carried out on a Beckman Avanti J-25 equipped with a JA-20 fixed-angle rotor, using 40 mL polypropylene screw-on cap tubes. The first ultracentrifugation step was performed on an Optima XPN-100 equipped with a TY45 Ti fixed angle rotor (Beckman Coulture Inc, Brea, CA, USA), using 50 mL polycarbonate bottle cap-assembly tubes. The sucrose cushion step was carried out on a benchtop Beckman Optima MAX-XP ultracentrifuge equipped with an MLS-50 swinging bucket rotor (Beckman Coulture Inc, Brea, CA, USA), using 7 mL polypropylene round-bottom tubes. The final washing step was conducted on the same benchtop ultracentrifuge, equipped with a TLA-55 fixed angle rotor, using 1.5 mL disposable polypropylene ultracentrifuge tubes.

All the procedures involving RBCs and RBC EV pellets resuspension, washing and transfer, were performed under sterile conditions. Non-sterile disposable tubes were sterilized by autoclave before

use. Non-disposable tubes were cold-sterilized in 10% H_2O_2 (Sigma-Aldrich Inc, St. Louis, MI, US) and rinsed multiple times with Milli-Q water (Merck-Millipore, Burlington, MA, US), according to the manufacturer's instructions. Metallic parts (e.g., MLS-50 rotor buckets and ultracentrifuge tube caps) were sanitized with 70% ethanol, rinsed multiple times with Milli-Q water and dried overnight in sterile conditions under UV-light. Unless specified otherwise, tubes were provided by Beckman Coulter.

Calcium chloride ($CaCl_2$), calcium ionophore, 30% hydrogen peroxide (H_2O_2) stock solution, and sucrose were purchased from Sigma-Aldrich (St. Louis, MO, USA). Disposable and cell culture plasticware for RBC-EV production were purchased from Sarstedt Ag & Co. KG, (Nümbrecht, Germany). Disposable and cell culture plasticware for MSCEV production were purchased from Greiner Bio-One (Kremsmünster, Austria), MEM-Alpha cell culture medium and supplements were purchased from Gibco (by ThermoFisher, Waltham, MA, USA). Sterile Phosphate Buffered Saline (PBS) was purchased from Lonza (Basel, Switzerland). 0.45 µm Primo PES syringe filters were purchased from Euroclone S.p.A., (Milan, Italy). Disposable, screw-cap, and bottle-cap assembly tubes for ultracentrifugation were purchased from Beckman Coulter Inc. (Brea, CA, USA). mPES hollow fiber filters (N04-E500-05-N) for MSC culture medium concentration were purchased from Repligen Corp (Waltham, MA, US), together with the necessary tubing and fittings. If not stated elsewhere, Milli-Q-grade water was used in all preparations.

The conditioned mesenchymal stem cells (MSC) medium was centrifuged at 800 x g for 30 minutes at 4°C (5804R centrifuge, A-4-44 swinging bucket rotor) to eliminate dead cells and cellular debris that may clog the filter, and then concentrated 20 times using a Tangential Flow Filtration device (KrosFlo by Repligen Corp, Waltham, MA, US) equipped with a 500kDa mPES hollow fiber filter (N04-E500-05-N by Repligen Corp). The device was put in a cell culture hood to ensure sterility conditions. The assembled filter (including tubing and fittings) was sanitized before use with 0.2M NaOH for 15 minutes in re-circulation mode, thoroughly rinsed with sterile water until reaching neutral pH, and checked for integrity according to the user's manual. The transmembrane pressure was set to 0.4 and automatically controlled through an automated backpressure valve, keeping the flowrate between 800 and 1000 ml/minute.

At the end of the concentration step, the filter was sanitized again with 0.2M NaOH, thoroughly washed with sterile water until pH neutralization, filled with a solution of 15% glycerol + 0.05% sodium azide and stored at 4°C. The permeate was discarded. The concentrated retentate (containing particles with a size > 30 nm) was centrifuged at 12'000 x g for 30 minutes to remove large EVs (on Avanti J-25, JA-20 fixed-angle rotor, 40 mL polypropylene screw-on cap tubes). The supernatant was collected and ultracentrifuged at 100'000 x g for 3 hours at 4°C (Optima XPN-100, TY45 Ti fixed

angle rotor, 50 mL polycarbonate bottle cap-assembly tubes). Each MSC-EV pellet was resuspended in 1 ml of PBS, diluted 6 times, aliquoted, characterized, and stored at -80°C until further use.

Non-sterile disposable tubes were sterilized by autoclave before use. Non-disposable tubes were cold-sterilized in 10% H_2O_2 and rinsed multiple times with Milli-Q water, according to the manufacturer's instructions.

S5.2 - Dynamic Light Scattering and ζ-Potential

The hydrodynamic diameter and surface charge of RBC EVs and MSC-EVs in Milli-Q water were evaluated through Dynamic Light Scattering and ζ-Potential, respectively, and reported in **Table S13**.

Table S13. Hydrodynamic diameter obtained from Dynamic Light Scattering and surface ζ-Potential values of RBC EVs and MSC EVs.

Sample	D_h (nm)	ζ-Potential (mV)
RBC-EVs	138 ± 27	-11.5 ± 0.2
MSC-EVs	166 ± 16	-25.5 ± 1.6

S5.3– Nanoparticle tracking analysis and protein content analysis

The hydrodynamic diameter and the concentration of RBC and MSC EVs resuspended in Milli-Q water were evaluated through Nanoparticle Tracking Analysis, while the total protein content was determined through Bicinchoninic Acid (BCA assay). The results are reported in **Table S14**.

Table S14. Hydrodynamic diameter and particle number obtained from Nanoparticle Tracking Analysis, and protein concentration obtained from BCA assay of RBC-EVs and MSC-EVs. For size, the modal diameter ± SEM of 3 replicates is shown. For particle number, the average of 3 measurements ± SEM is reported. For protein content, the average ± SD is shown.

Sample	D_h (nm)	Particles (p/ml)	Proteins (ug/uL)
RBC-EVs	161 ± 2.7 nm	2.47e+11 ± 1.19e+10	0.26 ± 0.03
MSC-EVs	187 ± 11.8 nm	3.16e+11 ± 2.65e+10	0.71 ± 0.02

S5.4 – Atomic Force Microscopy (AFM)

Table S15. The mechanical characterization of individual vesicles performed by measuring their stiffness via surface contact angle.

Sample	Average Contact Angle (°)	Standard Deviation (°)
DOPC	71	3
DOPC/POPC	84	4
POPC	82	3
POPC/DPPC	97	8
POPC/DSPC	107	8
RBC-EVs	82	7
MSC-EVs	101	5

References

(1) Haiss, W.; Thanh, N. T. K.; Aveyard, J.; Fernig, D. G. Determination of Size and Concentration of Gold Nanoparticles from UV-Vis Spectra. *Anal. Chem.* **2007**, *79* (11), 4215–4221. https://doi.org/10.1021/ac0702084.

(2) Liu, X.; Atwater, M.; Wang, J.; Huo, Q. Extinction Coefficient of Gold Nanoparticles with Different Sizes and Different Capping Ligands. **2007**, *58*, 3–7. https://doi.org/10.1016/j.colsurfb.2006.08.005.

(3) Schneider, C. A.; Rasband, W. S.; Eliceiri, K. W. NIH Image to ImageJ: 25 Years of Image Analysis. *Nat. Methods* **2012**, *9* (7), 671–675. https://doi.org/10.1038/nmeth.2089.

(4) Caselli, L.; Ridolfi, A.; Cardellini, J.; Sharpnack, L.; Paolini, L.; Brucale, M.; Valle, F.; Montis, C.; Bergese, P.; Berti, D. A Plasmon-Based Nanoruler to Probe the Mechanical Properties of Synthetic and Biogenic Nanosized Lipid Vesicles. *Nanoscale Horizons* **2021**, *6* (7), 543–550. https://doi.org/10.1039/D1NH00012H.

(5) Kotlarchyk, M.; Chen, S.-H. Analysis of Small Angle Neutron Scattering Spectra from Polydisperse Interacting Colloids. *J. Chem. Phys.* **1983**, *79* (5), 2461. https://doi.org/10.1063/1.446055.

(6) Menon, S. V. G.; Manohar, C.; Rao, K. S. A New Interpretation of the Sticky Hard Sphere Model. *J. Chem. Phys.* **1991**, *95* (12), 9186–9190. https://doi.org/10.1063/1.461199.

(7) Baxter, R. J. Percus–Yevick Equation for Hard Spheres with Surface Adhesion. *J. Chem. Phys.* **1968**, *49* (6), 2770–2774. https://doi.org/10.1063/1.1670482.

(8) Usman, M. W.; Pham, T. C.; Kwok, Y. Y.; Vu, L. T.; Ma, V.; Peng, B.; Chan, Y. S.; Wei, L.; Chin, S. M.; Azad, A.; He, A. B. L.; Leung, A. Y. H.; Yang, M.; Shyh-Chang, N.; Cho, W. C.; Shi J.; Le, M. T. N. Efficient RNA Drug Delivery Using Red Blood Cell Extracellular Vesicles, *Nat. Commun.*, 2018, **9**, Article number: 2359. https://doi.org/10.1038/s41467-018-04791-8.
